# Design and Synthesis of an ^18^F-Labeled Version of Phenylethyl Orvinol ([^18^F]FE-PEO) for PET-Imaging of Opioid Receptors

**DOI:** 10.3390/molecules171011554

**Published:** 2012-09-28

**Authors:** János Marton, Gjermund Henriksen

**Affiliations:** 1 ABX Advanced Biochemical Compounds Biomedizinische Forschungsreagenzien GmbH, Heinrich-Glaeser-Strasse 10-14, D-01454 Radeberg, Germany; Email: marton@abx.de; 2 Oslo PET-Centre, Norsk Medisinsk Syklotronsenter AS, N-0027 Oslo, Norway; 3 Institute of Basic Medical Sciences, Group of Pharmaceutical Radiochemistry, University of Oslo, Postboks 1110 Blindern, N-0317 Oslo, Norway

**Keywords:** opioid receptors, PET, agonist, ^18^F-fluorination, automated radiosynthesis

## Abstract

The semisynthetic oripavine derivative phenethyl orvinol (PEO), a full agonist at opioid receptors (OR), is an attractive structural motif for developing ^18^F-labeled PET tracers with a high degree of sensitivity for competition between endogenous and exogenous OR-ligands. The target cold reference compound 6-*O*-(2-fluoroethyl)-6-*O*-desmethylphenylethyl orvinol (FE-PEO) was obtained via two separate reaction routes. A three-step synthesis was developed for the preparation of a tosyloxyethyl precursor (TE-TDPEO), the key precursor for a direct, nucleophilic radiofluorination to yield [^18^F]FE-PEO. The developed radiosynthesis provides the target compound in relevantly high yield and purity, and is adaptable to routine production.

## Abbreviations

MOP: μ-opioid receptor
KOP: κ-opioid receptor
DOP: δ-opioid receptor
HMBC: heteronuclear multiple bond correlations
HMQC: heteronuclear multiple quantum correlations
L-Selectride: lithium tri-*sec*-butylborohydride
Ph: phenyl group
TBDPS: *tert*-butyldiphenylsilyl group
TBDMS: *tert*-butyldimethylsilyl group
Tr: trityl group = triphenylmethyl group
DMAP: 4-dimethylaminopyridine
DIPEA: *N*-ethyldiisopropylamine
TDPEO: 3-*O*-trityl-6-*O*-desmethyl-phenylethyl orvinol
TBDPSOE-TDPEO: 6-*O*-(2-*tert*-butyldiphenylsilyloxyethyl)-3-*O*-trityl-6-*O*-desmethyl-phenylethyl orvinol
TBDMSE-TDPEO: 6-*O*-(2-*tert*-butyldimethylsilyloxyethyl)-3-*O*-trityl-6-*O*-desmethyl-phenylethyl orvinol
HE-TDPEO: 6-*O*-(2-hydroxyethyl)-3-*O*-trityl-6-*O*-desmethyl-phenylethyl orvinol
TE-TDPEO: 6-*O*-(2-tosyloxyethyl)-3-*O*-trityl-6-*O*-desmethyl-phenylethyl orvinol
FE-TDPEO: 6-*O*-(2-fluoroethyl)-3-*O*-trityl-6-*O*-desmethyl-phenylethyl orvinol
FE-PEO: 6-*O*-(2-fluoroethyl)-6-*O*-desmethyl-phenylethyl orvinol
DPET: 6-*O*-desmethyl-phenylethyl thevinol
FE-DPET: 6-*O*-(2-fluoroethyl)-6-*O*-desmethyl-phenylethyl thevinol
E-DPET: 6-*O*-ethyl-6-*O*-desmethyl-phenylethyl thevinol
PEO: (20*R*)-phenylethyl orvinol
DPEO: 6-*O*-desmethyl-phenylethyl orvinol
TDPEO: 6-*O*-trityl-6-*O*-desmethyl-phenylethyl orvinol

## 1. Introduction

The expression and function of opioid receptors (ORs) are known to be altered in numerous pathophysiological processes [1,2]. Currently, three well-defined OR-subtypes, MOP, KOP and DOP, are known to be expressed in the central nervous system and in the periphery. The effect of affinity and subclass selectivity on the sensitivity of a tracer for detecting altered availability from endogenous and exogenous opioid receptor binding compounds have been addressed [1,2,3,4]. In contrast, the effects of the intrinsic activity of the tracer on these parameters have so far not been systematically studied. The ORs belong to the superfamily of G-protein-coupled receptors and it is hypothesized that these receptors can exist in two different conformational states *in vivo*, *i.e.*, G protein coupled and uncoupled. Results from *in vitro* studies suggest that agonists bind with higher affinity to the receptor when in the coupled state, whilst antagonists bind with the same affinity to the two states [5].

The results from our previous investigations [6,7,8] have shown that 3-*O*-trityl-6-*O*-desmethyl-phenylethyl orvinol (TDPEO, **1**) is a suitable precursor for the synthesis of [6-*O*-methyl-^11^C]PEO, an orvinol μ- and κ-agonist radioligand. To benefit from the practical advantages from the longer physical half-life of ^18^F, an ^18^F-labeled PEO-derivative is attractive. In this work we describe the strategy and practical route to a precursor and reference standard for an ^18^F-fluoroalkyl labelled PEO derivative: 6-*O*-(2-[^18^F]fluoroethyl)-6-*O*-desmethylphenylethyl orvinol ([^18^F]FE-PEO, [^18^F]**3**).

## 2. Results and Discussion

### 2.1. Chemistry

#### 2.1.1. Synthesis of FE-PEO (**3**) from 3-*O*-Trityl Protected Phenylethyl Orvinol

**Scheme 1:**
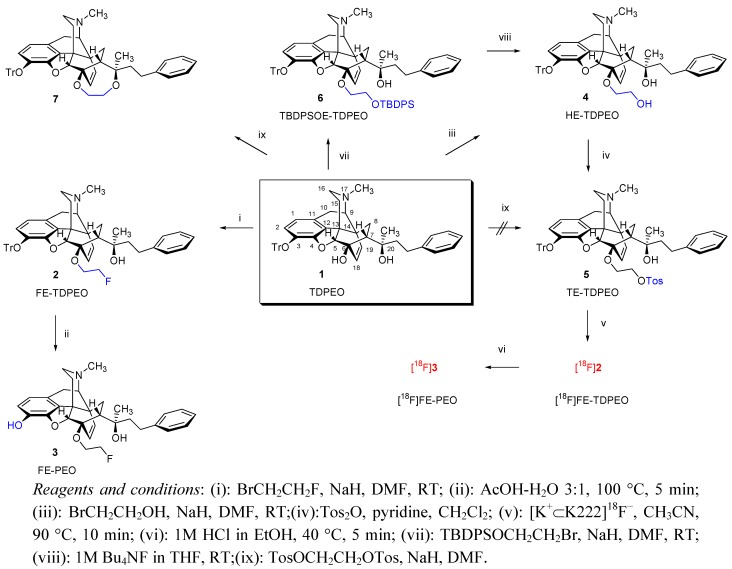
Synthesis of 6-*O*-(2-fluoroethyl)-6-*O*-desmethylphenylethyl orvinol (**3**, FE-PEO) and TE-TDPEO (**5**) for use as a precursor for radiosynthesis of [^18^F]FE-PEO ([^18^F]**3**).

The synthesis of 6-*O*-(2-fluoroethyl)-6-*O*-desmethylphenylethyl orvinol (FE-PEO, **3**) was accomplished by two different reaction routes. The first sequence (route A: **1** → **2** → **3**) is depicted in Scheme 1. The starting material 3-*O*-trityl-6-*O*-desmethylphenylethyl orvinol (TDPEO, **1**) was obtained from the poppy alkaloid thebaine in five steps, as described previously [7]. In brief, the *endo*-etheno-bridged, rigid structural motif was obtained from a Diels-Alder (DA) cycloaddition of thebaine and methyl vinyl ketone. Applied to morphinan-6,8-dienes with unsymmetrical dienophiles, this reaction can afford eight isomers [9]. The addition of methyl vinyl ketone to thebaine occurs regio- and stereoselectively on the β-face and results exclusively in a mixture of 7-acetyl derivatives (7α-acetyl:7β-acetyl = 98:2) [10]. The 7α-acetyl derivative (thevinone) was reacted with 2-phenylethyl-magnesium bromide, which yielded a complex product mixture comprising two diastereomeric tertiary alcohols (normal Grignard reaction products), two diastereomeric secondary carbinols (Grignard reduction, beta-hydrogen transfer), as well as an additional byproduct which contains a [5,6,7]cyclopropane ring in addition to a phenolic hydroxyl group in position 4, the latter being a result of a base-catalysed rearrangement [11]. The reaction took place with high degree of stereoselectivity in accordance with Cram’s rule, and resulted in the tertiary alcohol **8** with absolute 20*R* configuration as the main product (62%). 3-*O*-Demethylation of 20*R*-phenylethyl thevinol (**8**) afforded 20*R*-phenylethyl orvinol (PEO, **12**). Subsequently, **12** was 6-*O*-demethylated to yield 6-*O*-desmethyl-PEO (DPEO, **13**). Finally, the phenolic function in the position 3 was protected via the introduction of a trityl protection group (TDPEO, **1**).

For preparation of FE-PEO (**3**), TDPEO (**1**) was first selectively fluoroalkylated by treatment with 2-fluoroethyl bromide in *N*,*N*-dimethylformamide in the presence of sodium hydride, which resulted in 6-*O*-(2-fluoroethyl)-6-*O*-desmethyl-3-*O*-tritylphenylethyl orvinol (FE-TDPEO, **2**). Subsequent cleavage of the 3-*O*-trityl bond with 60% acetic acid [12] afforded 6-*O*-(2-fluoroethyl)-6-*O*-desmethyl-phenylethyl orvinol (FE-PEO, **3**) in good overall yield (49% from **1**) and purity > 95%. In the HMBC spectrum of FE-PEO (**3**), the correlation between the 18-H of the 6,14-etheno bridge and CH_2_CH_2_F (88.3) as well as between CH_2_CH_2_F/CH_2_CH_2_F and C-5 (98.9) were observed, which verified that 6-*O*-substitution had occurred. On the other hand, the correlation between 20-OH (4.81) and C-20 (74.9), C7 (47.5), PhCH_2_CH_2_ (42.9) firmly establishes that the 20-OH functionality is retained.

#### 2.1.2. Synthesis of FE-PEO (**3**) from 20*R*-Phenylethyl thevinol

**Scheme 2:**
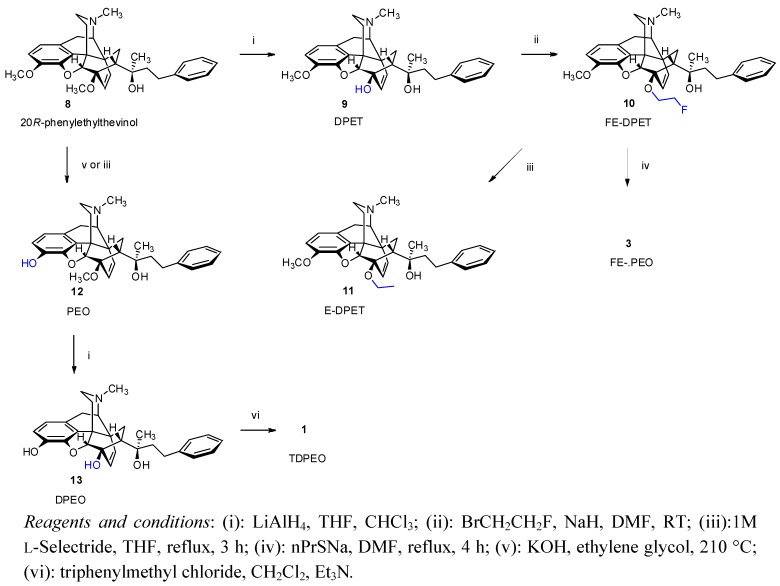
Synthesis of 6-*O*-(2-fluoroethyl)-6-*O*-desmethyl-phenylethyl orvinol (**3**, FE-PEO) from 20*R*-phenylethyl thevinol.

The alternative sequence (route B: **8** → **9** → **10** → **3**) is shown in Scheme 2, using 20*R*-phenylethyl thevinol (**8**) as the starting point. Here, **8** was 6-*O*-demethylated according to the method described by Luthra *et al*. [13] to yield 6-*O*-desmethyl-phenylethyl thevinol (DPET, **9**). Fluoroethylation of the 6-OH tertiary alcohol (2-fluoroethyl bromide, NaH, DMF) led to 6-*O*-(2-fluoroethyl)-6-*O*-desmethyl-phenylethyl thevinol (FE-DPET, **10**).

The 3-*O*-demethylation of morphine alkaloids has proven difficult in general (tedious work-ups, poor chemical yields). With some thevinols this reaction was readily accomplished by the use of L-Selectride [14] (lithium tri-*sec*-butylborohydride). This reagent was first reported to be an efficient 3-*O*-demethylating agent for the morphine alkaloids (e.g., thebaine → oripavine; oxycodone indole → oxymorphindole; 20-methylthevinol → 20-methylorvinol ) by Coop *et al*. [14]. This method is proven essential for the preparation of 6,14-ethenomorphinans with tertiary alcohol function in the position-20 (thevinol → orvinol conversion).

In addition, application of boron tribromide or other acidic agents for the 3-*O*-demethylation of thevinols is unsuitable, in particular, these substrates are prone to acid-catalyzed dehydration, enol ether hydrolysis and rearrangement, e.g., to anhydro-20-alkylthevinols, to 14-alkenylcodeinones or to 5,14-bridged thebainone derivatives [15]. In favourable contrast, L-Selectride is efficient and generally applicable to 3-*O*-demethylation of morphine alkaloids [14] under mild conditions, and the desired 3-phenolic compounds were obtained in higher yield and purity relative to that obtained by the application of other reagents (e.g., potassium hydroxide, diethylene glycol, 210 °C [16,17]).

In the present work, 6-*O*-(2-fluoroethyl)-6-*O*-desmethylphenylethyl thevinol (FE-DPET, **10**) was refluxed with three equivalents of L-Selectride in tetrahydrofuran. 6-*O*-Ethyl-6-*O*-desmethyl-phenylethyl thevinol (E-DPET, **11**) was isolated as the sole product after 3 h reaction time, demonstrating that reductive defluorination reaction preferentially took place over *O*-demethylation. In contrast, *O*-demethylation of FE-DPET (**10**) was readily accomplished by the use of sodium propanethiolate in DMF to yield FE-PEO (**3**). In summary, L-Selectride was successfully used in the present study for the preparation of PEO (**12**) from 20*R*-phenylethyl thevinol (**8**), but in the case of the newly synthesized 6-*O*-(2-fluoroalkyl)thevinols the application was limited (reductive defluorination occurred instead of the desired 3-*O*-demethylation).

#### 2.1.3. Synthesis of the Precursor TE-TDPEO (**5**)

We initially aimed at developing a method for preparation of tosylated precursor TE-TDPEO (**5**) (Scheme 1) starting from TDPEO (**1**). However, a direct introduction of the tosyloxyethyl group proved difficult (solvent: DMF or acetonitrile; base: NaH, KOtBu). Alkylation of **1** was successfully accomplished by the use of 2-bromoethanol at room temperature in DMF, in the presence of sodium hydride. The target compound 6-*O*-(2-hydroxyethyl)-6-*O*-desmethyl-3-*O*-tritylphenylethyl orvinol (HE-TDPEO, **4**) was isolated after 48 h, in only 19% yield and starting material **1** (37%) was recovered. Attempts to promote alkylation at higher temperature or using a large excess of reagent resulted in decomposition.

**Scheme 3:**
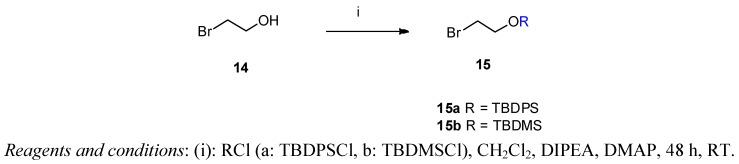
Preparation of the silyl protected 2-bromoethanol reagents.

The preferred strategy for introducing the desired functionality at the 6-*O*-position comprised three conversions. Firstly, 2-bromoethanol was allowed to react at RT with the corresponding silylated reagent [*tert*-butyldiphenylsilyl chloride (TBDPSCl) or *tert*-butyldimethylsilyl chloride (TBDMSCl)] in the presence of DIPEA and DMAP to produce the silyl-protected agent (compounds **15a** and **15b**) (Scheme 3). Secondly, the *tert*-butyldiphenylsilyl derivate (TBDPSOE-TDPEO, **6**) was prepared from TDPEO (**1**) by means of the reaction with 2.33 equivalents of TBDPSOCH_2_CH_2_Br (**15a**) at RT (81% yield). The subsequent selective cleavage of the silyl protecting group with tetrabutylammonium fluoride in THF [18] provided HE-TDPEO (**4**) in 75% yield. Finally, the resulting primary alcohol was tosylated by *p*-toluenesulfonic anhydride to give the desired new TE-TDPEO (**5**) precursor (overall yield: 32%).

1D (^1^H-, ^13^C-, ^19^F), 2D NMR techniques and ESI-MS were used for the identification of the prepared compounds. ^1^H and ^13^C-NMR assignments were obtained from ^1^H-^1^H correlation spectroscopy (COSY), 2D heteronuclear ^1^H-^13^C multiple quantum correlations experiments (HMQC) and heteronuclear multiple bond correlations (HMBC) and facilitated by previously published NMR studies on various 6,14-ethenomorphinan derivatives [13,19,20,21,22]. NMR spectral assignments are presented in the Experimental Section.

#### 2.1.4. Radiolabeling

[^18^F]FE-PEO ([^18^F]**3**) was prepared from TE-TDPEO (**5**) in a two-step, one-pot ^18^F-fluorination. TE-TDPEO (**5**) was reacted with activated [^18^F]fluoride, followed by acidic removal of the 3-*O*-trityl group [7]. After preparative HPLC and work-up, [^18^F]FE-PEO was produced in 35 ± 8% isolated preparative yield (not optimized), a specific activity of 1490-3458 mCi/μmol, and a radiochemical purity of >99% *at end-of-synthesis*, (~42 min after *end-of-bombardment*). The isolated product ([^18^F]**3**) co-eluted with an authentic reference of FE-PEO (**3**) on two independent analytical HPLC-systems (see Experimental Section).

## 3. Experimental

### 3.1. General Methods

Reagents and solvents were purchased from major commercial suppliers and were used without further purification. Melting points were measured with a Büchi-535 instrument and the data are uncorrected. ^1^H-NMR and ^13^C-NMR spectra were obtained with a Bruker 500 spectrometer at 20 °C in CDCl_3_. Column chromatography was performed on Kieselgel 60 Merck 1.09385 (0.040–0.063 mm). TLC was accomplished on Macherey-Nagel Alugram^®^ Sil G/UV_254_ 40 × 80 mm aluminum sheets [0.25 mm silica gel with fluorescent indicator] with the following eluent systems (each v/v): [A]: chloroform-methanol 95:5; [B]: ethyl acetate-methanol 8:2; [C]: hexane-ethyl acetate 7:3; [D]: hexane-ethyl acetate 1:1. The spots were visualized with a 254 nm UV lamp or with 5% phosphomolybdic acid in ethanol.

### 3.2. Chemistry

*(5R,6R,7R,9R,13S,14R,20R)-(5α,7α)-4,5-epoxy-6-(2-fluoroethoxy)-α,17-dimethyl-α-(2-phenylethyl)-3-triphenylmethoxy-6,14-ethenomorphinan-7-methanol* (**2**, FE-TDPEO). Sodium hydride (240 mg, 10 mmol) was suspended in dry *N*,*N*-dimethylformamide (6 mL) under argon. A solution of TDPEO [7] (**1**, CAS RN: [1187551-69-4], 700 mg, 1 mmol) in dry *N*,*N*-dimethylformamide (6 mL) was added dropwise in and the suspension was stirred for 15 min. 2-Bromofluoroethane (175 μL, 300 mg, 2.33 mmol) was added via syringe and the mixture was stirred for 48 h. The product mixture was poured into water (30 mL). The suspension was extracted with chloroform (4 × 50 mL). The combined organic phases were dried (Na_2_SO_4_) and concentrated in a rotary evaporator under reduced pressure. The crude product was purified by column chromatography on silica gel [150 g, eluent system: hexane-ethyl acetate 7:3 (v/v)]. The product was dried under vacuum to yield 480 mg (64%) of **2** as yellowish foam. R_f_ [chloroform-methanol 9:1] = 0.95; R_f_ [C] 0.35; R_f_ [D] = 0.81. ^1^H-NMR (CDCl_3_) δ = 7.32–7.36 (m, 6H, o-Tr); 7.20–7.27 (m, 9H, Tr(*m,p*)); 7.12–7.20 (m, 5H, PhCH_2_CH_2_); 6.28 (d, ^2^*J*_2,1_ = 8.1 Hz, 1H, 2-H); 6.12 (d, ^2^*J*_1,2_ = 8.1 Hz, 1H, 1-H); 5.74 (d, ^2^*J*_18,19_ = 8.9 Hz, 1H, 18-H); 5.29 (d, ^2^*J*_19,18_ = 8.9 Hz, 1H, 19-H); 4.81 (br s, 1H, 20-OH); 4.32–4.57 (m, 2H, 6-*O*-CH_2_CH_2_F); 4.24 (d, ^4^*J*_5β,18_ = 1.0 Hz, 1H, 5β-H); 3.91–4.14 (m, 2H, 6-*O*-CH_2_CH_2_F); 3.05 (d, ^2^*J*_10β,10α_ = 18.7 Hz, 1H, 10β-H); 2.98 (d, ^3^*J*_9α,10α_ = 6.4 Hz, 1H, 9α-H); 2.78 (m, 1H, 8β-H); 2.70–2.76 (m, 2H, PhCH_2_CH_2_); 2.39 (dd, ^2^*J*_16eq,16ax_ = 12.1 Hz, ^3^*J*_16eq,15ax_ = 4.9 Hz, 1H, 16-H_eq_); 2.26 (s, 3H, NCH_3_); 2.23 (m, 1H, 16-H_ax_); 2.18 (m, 1H, 10α-H); 1.94 (app t, 1H, ^3^*J*_7β,8α_ = 8.4 Hz, 7β-H); 1.77 (td, ^2^*J*_15ax,15eq_ = 13.9 Hz, ^3^*J*_15ax,16ax_ = 12.9 Hz, ^3^*J*_15ax,16eq_ = 5.4 Hz, 1H, 15H_ax_); 1.65 (m, 1H, 15-H_eq_); 1.53–1.58 (m, 2H, PhCH_2_CH_2_); 1.03 (s, 3H, 20-CH_3_); 0.74 (dd, ^2^*J*_8α,8β_ = 13.0 Hz, ^3^*J*_8α,7β_ = 8.4 Hz, 1H, 8α-H). ^13^C-NMR δ = 152.5 (C4); 144.2 (TrC1); 143.2 (C3); 137.3 (Ph-C1); 135.4 (C12); 133.5 (C19); 130.2 (C18); 125.3 (C11); 129.4 (*o*CTr); 128.4 (Ph-C3,5); 128.2 (Ph-2,6); 127.3 (*m*CTr); 127.2 (*p*CTr); 125.5 (Ph-C4); 122.8 (C1); 118.3 (C2); 97.9 (C5); 91.5 (TrCO); 84.2 (C6); 83.1 (d, *J* = 169.6 Hz, 6-*O*-CH_2_CH_2_F); 74.5 (C20); 66.7 (d, *J* = 18.8 Hz, 6-*O*-CH_2_CH_2_F); 59.7 (C9); 47.4 (C7); 46.9 (C13); 45.2 (C16); 43.4 (NCH_3_); 42.8 (PhCH_2_CH_2_); 42.9 (C14); 33.5 (C15); 30.6 (C8); 29.1 (PhCH_2_CH_2_); 23.1 (20-CH_3_); 22.3 (C10). ^19^F-NMR δ = −224.5 ESI-MS *m/z*: 748 [M+1]^+^ C_50_H_50_FNO_4_ (747.93).

*(5R,6R,7R,9R,13S,14R,20R)-(5α,7α)-4,5-epoxy-6-(2-fluoroethoxy)-3-hydroxy-α,17-dimethyl-α-(2-phenylethyl)-6,14-ethenomorphinan-7-methanol* (**3**, FE-PEO). Method A: 6-*O*-(2-Fluoroethyl)-6-*O*-desmethyl-3-*O*-tritylphenylethyl orvinol (**2**, FE-TDPEO, 430mg, 0.57 mmol) was dissolved in a mixture of acetic acid (30 mL) and water (10 mL). The solution was stirred at 100 °C for 5 min. Analytical TLC of the product mixture after 5 min reaction time showed absence of starting material **2**. For work-up, the solution cooled to room temperature and thereafter poured into ice-water (50 mL). The pH of the mixture was adjusted to 9 with NH_4_OH. The suspension was extracted with chloroform (4 × 50 mL). The combined organic phase was dried (Na_2_SO_4_) and the solvent was evaporated. The residue was purified by column chromatography on silica gel [150 g, eluent system: hexane-ethyl acetate 1:1 (v/v)]. The product was dried under vacuum (3 × 10^−1^ mbar, 16 h). Yield: 226 mg (78%) **3** as a yellow solid. mp. 139–141 °C. R_f_ [chloroform-methanol 9:1] = 0.82; R_f_ [C] 0.11; R_f_ [D] = 0.34. Method B: Sodium hydride (190 mg, 7.91 mmol) was suspended in *N*,*N*-dimethylformamide (5 mL) under argon atmosphere. Propane-1-thiol (100 μL, 1.1 mmol) in *N*,*N*-dimethylformamide (2 mL) was added and the mixture was stirred for 15 min. A solution of 6-*O*-(2-fluoroethyl)-6-*O*-desmethyl-phenylethyl thevinol (**10**, FE-DPET, 340 mg, 0.65 mmol) in *N*,*N*-dimethylformamide (4 mL) was added dropwise and the reaction mixture was refluxed for 4 h. The mixture was then poured into ice-water (30 g), thereafter acidified to pH = 2 with a 1 M HCl solution and finally extracted with diethyl ether (2 × 20 mL). The aqueous phase was alkalised with NH_4_OH and extracted with dichloromethane-methanol 9:1 (v/v) (5 × 50 mL). The combined organic phases were dried (Na_2_SO_4_) and the solvent was removed under reduced pressure to yield a brownish solid. The crude product was purified by column chromatography on silica gel (120 g) as described above. Yield 195 mg (59%). ^1^H-NMR (CDCl_3_) δ = 7.13–7.27 (m, 5H, PhCH_2_CH_2_); 6.57 (d, ^2^*J*_2,1_ = 8.1 Hz, 1H, 2-H); 6.46 (d, ^2^*J*_1,2_ = 8.1 Hz, 1H, 1-H); 5.89 (d, ^2^*J*_18,19_ = 8.9 Hz, 1H, 18-H); 5.43 (d, ^2^*J*_19,18_ = 8.9 Hz, 1H, 19-H); 4.81 (br s, 1H, 20-OH); 4.55 (br s, 1H, 3-OH); 4.50–4.68 (m, 2H, 6-*O*-CH_2_CH_2_F); 4.57 (d, ^4^*J*_5β,18_ = 0.8 Hz, 1H, 5β-H); 4.14–4.38 (m, 2H, 6-*O*-CH_2_CH_2_F); 3.17 (d, ^2^*J*_10β,10α_ = 18.5 Hz, 1H, 10β-H); 3.08 (d, ^3^*J*_9α,10α_ = 6.4 Hz, 1H, 9α-H); 2.86 (dd, 1H, ^2^*J*_8β,8α_ = 12.7 Hz, ^3^*J*_8β,7β_ = 9.1 Hz, 8β-H); 2.71–2.81 (m, 2H, PhCH_2_CH_2_); 2.47 (dd, ^2^*J*_16eq,16ax_ = 11.9 Hz, ^3^*J*_16eq,15ax_ = 4.8 Hz, 1H, 16-H_eq_); 2.36 (m, 1H, 16-H_ax_); 2.32 (s, 3H, NCH_3_); 2.31 (m, 1H, 10α-H); 2.05 (app t, 1H, ^3^*J*_7β,8α_ = 8.6 Hz, 7β-H); 1.91 (td, ^2^*J*_15ax,15eq_ = 13.6 Hz, ^3^*J*_15ax,16ax_ = 12.7 Hz, ^3^*J*_15ax,16eq_ = 5.4 Hz, 1H, 15H_ax_); 1.80 (dd, 1H, ^2^*J*_15eq,16ax_ = 13.6 Hz, ^3^*J*_15eq,16ax_ = 2.4 Hz, 15-H_eq_); 1.55-1.73 (m, 2H, PhCH_2_CH_2_); 1.06 (s, 3H, 20-CH_3_); 0.82 (dd, ^2^*J*_8α,8β_ = 12.7 Hz, ^3^*J*_8α,7β_ = 8.3 Hz, 1H, 8α-H). ^13^C-NMR δ = 146.6 (C4); 143.1 (C3); 137.3 (Ph-C1); 135.8 (C12); 133.9 (C19); 128.5 and 128.2 (Ph-C3,5 and Ph-2,6); 127.8 (C11); 125.5 (Ph-C4); 125.0 (C18); 119.9 (C1); 116.3 (C2); 98.9 (C5); 84.4 (C6); 83.0 (d, *J* = 169.9 Hz, 6-*O*-CH_2_CH_2_F); 74.9 (C20); 66.7 (d, *J* = 18.8 Hz, 6-*O*-CH_2_CH_2_F); 59.8 (C9); 47.5 (C13); 47.4 (C7); 45.4 (C16); 43.5 (NCH_3_); 43.0 (C14); 42.9 (PhCH_2_CH_2_); 33.5 (C15); 30.6 (C8); 29.1 (PhCH_2_CH_2_); 23.2 (20-CH_3_); 22.3 (C10). ^19^F-NMR δ = −224.5 ESI-MS *m/z*: 506 [M+1]^+^ C_31_H_36_FNO_4_ (505.62).

*(5R,6R,7R,9R,13S,14R,20R)-(5α,7α)-4,5-Epoxy-6-(2-hydroxyethoxy)-α,17-dimethyl-α-(2-phenylethyl)-3-triphenylmethoxy-6,14-ethenomorphinan-7-methanol* (**4**, HE-TDPEO). Method A: Sodium hydride (240 mg, 10 mmol) was suspended in dry *N*,*N*-dimethylformamide (6 mL) and cooled to 0 °C. TDPEO [7] (**1**, 700 mg, 1 mmol) was added and the suspension stirred for 15 min. 2-Bromethanol (165 μL, 2.33 mmol) was added dropwise and the reaction mixture was stirred at room temperature for 48 h. The product mixture was added to cold water (50 mL) and the resulting suspension was extracted with dichloromethane (5 × 80 mL). The combined organic phases were washed with brine (100 mL), dried (Na_2_SO_4_) and the solvent was evaporated under reduced pressure. The crude product was purified by column chromatography on silica gel [150 g, eluent: hexane-ethyl acetate 7:3 (v/v)]. Fraction 1 (**1**): 259 mg (37%) Fraction 2 (**4**): 143 mg (19%). Method B: TBDPSOE-TDPEO (**6**, 670 mg, 0.68 mmol) was dissolved in dry tetrahydrofuran (25 mL) under argon atmosphere. 0.87 mL (0.87 mmol) of a 1 M solution of tetrabutylammonium fluoride in tetrahydrofuran was added to the mixture. The yellowish solution was stirred at room temperature. The starting material was absent after 4 h according to TLC. The solvent was removed by rotary evaporation. Water (70 mL) was added to the residue. After extraction with dichloromethane (5 × 100 mL), the combined organic phases were dried (Na_2_SO_4_) and the solvent was evaporated under vacuum. The product was purified by column chromatography on silica gel (170 g, eluent: hexane-ethyl acetate 7:3 to 1:1 (v/v). Yield: 384 mg (75%) white crystalline product (**4**); mp. 196–197 °C R_f_ [A] = 0.76; R_f_ [B] = 0.80; R_f_ [C] = 0.11; R_f_ [D] = 0.28. ^1^H-NMR (CDCl_3_) δ = 7.33–7.37 (m, 6H, Tr(*o*)); 7.21–7.27 (m, 9H, Tr(*m,p*)); 7.13–7.21 (m, 5H, 20-CH_2_CH_2_Ph); 6.29 (d, ^2^*J*_2,1_ = 8.4 Hz, 1H, 2-H); 6.12 (d, ^2^*J*_1,2_ = 8.4 Hz, 1H, 1-H); 5.78 (d, ^2^*J*_18,19_ = 8.9 Hz, 1H, 18-H); 5.28 (d, ^2^*J*_19,18_ = 8.9 Hz, 1H, 19-H); 5.01 (br s, 1H, 20-OH); 4.23 (d, ^4^*J*_5β,18_ = 1.0 Hz, 1H, 5β-H); 3.81–4.08 (m, 2H, 6-*O*-CH_2_CH_2_OH); 3.64–3.77 (m, 2H, 6-*O*-CH_2_CH_2_OH); 3.04 (d, ^2^*J*_10β,10α_ = 18.7 Hz, 1H, 10β-H); 2.98 (d, ^3^*J*_9α,10α_ = 6.1 Hz, 1H, 9α-H); 2.77 (m, 1H, 8β-H); 2.71–2.76 (m, 2H, 20-CH_2_CH_2_Ph); 2.39 (dd, ^2^*J*_16eq,16ax _ = 12.1 Hz, ^3^*J*_16eq,15ax_ = 5.0 Hz, 1H, 16-H_eq_); 2.26 (s, 3H, NCH_3_); 2.21 (m, 1H, 16-H_ax_); 2.18 (m, 1H, 10α-H); 1.94 (app t, ^3^*J*_7β,8α_ = 8.1 Hz, 1H, 7β-H); 1.77 (td, ^2^*J*_15ax,15eq_ = 13.8 Hz, ^3^*J*_15ax,16ax _ = 12.8 Hz, ^3^*J*_15ax,16eq_ = 5.5 Hz, 1H, 15-H_ax_); 1.71 (br s, 1H, 6-*O*-CH_2_CH_2_OH); 1.65 (m, 1H, 15-H_eq_); 1.51–1.58 (m, 2H, 20-CH_2_CH_2_Ph); 1.03 (s, 3H, 20-CH_3_); 0.74 (dd, ^2^*J*_8α,8β_ = 13.1 Hz, ^3^*J*_8α,7β_ = 8.1 Hz, 1H, 8α-H). ^13^C-NMR δ = 152.4 (C4); 144.1 (TrC1); 143.2 (C3); 137.3 (Ph-C1); 135.2 (C12); 133.6 (C19); 130.2 (C18); 129.4 (*o*CTr); 128.4 (Ph-C3,5); 128.3 (Ph-C2,6); 127.3 (*m*CTr); 127.2 (*p*CTr); 125.6 (C11); 125.5 (Ph-C4); 122.7 (C1); 118.4 (C2); 98.0 (C5); 91.5 (TrCO); 81.1 (C6); 74.7 (C20); 68.6 (6-*O*-CH_2_CH_2_OH); 62.6 (6-*O*-CH_2_CH_2_OH); 59.7 (C9); 47.3 (C7); 46.8 (C13); 45.3 (C16); 43.5 (NCH_3_); 43.0 (20-CH_2_CH_2_Ph); 42.7 (C14); 33.4 (C15); 30.6 (C8); 29.1 (20-CH_2_CH_2_Ph); 23.5 (20-CH_3_); 22.3 (C10). ESI-MS *m/z*: 746 [M+1]^+^ C_50_H_51_NO_5_ (745.94).

*(5R,6R,7R,9R,13S,14R,20R)-(5α,7α)-4,5-Epoxy-α,17-dimethyl-α-(2-phenylethyl)-6-(2-(4-toluene-sulfonyloxy)ethoxy)-3-triphenylmethoxy-6,14-ethenomorphinan-7-methanol* (**5**, TE-TDPEO). 6-*O*-(2-Hydroxyethyl)-TDPEO (**4**, 364 mg, 0.487mmol) was dissolved in dry dichloromethane (12 mL). The solution was cooled to 0 °C and pyridine (160 μL) was added. After stirring for 15 min, toluenesulfonic anhydride (540 mg, 1.65 mmol) was added in small portions. The reaction mixture was stirred for 4 h at room temperature. The product mixture was poured into water (70 mL) and the resulting suspension was extracted with dichloromethane (6 × 70 mL). The combined organic phases were dried (Na_2_SO_4_) and the solvent was removed under vacuum. The product was purified by flash chromatography [silica gel: 175 g, eluent: hexane-ethyl acetate 7:3 (v/v)]. Yield: 240 mg (54%) yellowish oil. R_f_ [A] = 0.76; R_f_ [hexane-ethyl acetate-NH_4_OH 7:3:0.1] = 0.13; R_f_ [hexane-ethyl acetate-NH_4_OH 1:1:0.1] = 0.59; R_f_ [chloroform-methanol 100:2] = 0.54. ^1^H-NMR (CDCl_3_) δ = 7.80 (d, *J* = 8.2 Hz, 2H, Tos-2,6); 7.32 (d, *J* = 8.2 Hz, 2H, Tos-3,5); 7.29–7.34 (m, 6H, Tr(*o*)); 7.12–7.27 (m, 5H, 20-CH_2_CH_2_Ph); 7.13–7.21 (m, 9H, Tr(*m,p*)); 6.27 (d, ^2^*J*_2,1_ = 8.2 Hz, 1H, 2-H); 6.11 (d, ^2^*J*_1,2_ = 8.2 Hz, 1H, 1-H); 5.66 (d, ^2^*J*_18,19_ = 8.9 Hz, 1H, 18-H); 5.27 (d, ^2^*J*_19,18_ = 8.9 Hz, 1H, 19-H); 4.38 (br s, 1H, 20-OH); 4.05 (d, ^4^*J*_5β,18_ = 1.0 Hz, 1H, 5β-H); 4.01–4.11 (m, 2H, 6-*O*-CH_2_CH_2_OTos); 3.86–4.00 (m, 2H, 6-*O*-CH_2_CH_2_OTos); 3.03 (d, ^2^*J*_10β,10α_ = 18.7 Hz, 1H, 10β-H); 2.96 (d, ^3^*J*_9α,10α_ = 6.4 Hz, 1H, 9α-H); 2.75 (m, 1H, 8β-H); 2.69–2.75 (m, 2H, 20-CH_2_CH_2_Ph); 2.42 (s, 3H, TosCH_3_); 2.37 (dd, ^2^*J*_16eq,16ax _ = 12.0 Hz, ^3^*J*_16eq,15ax_ = 4.8 Hz, 1H, 16-H_eq_); 2.25 (s, 3H, NCH_3_); 2.20 (m, 1H, 16-H_ax_); 2.17 (m, 1H, 10α-H); 1.83 (app t, ^3^*J*_7β,8α_ = 8.4 Hz, 1H, 7β-H); 1.71 (td, ^2^*J*_15ax,15eq_ = 13.8 Hz, ^3^*J*_15ax,16ax_ = 12.9 Hz, ^3^*J*_15ax,16eq_ = 5.4 Hz, 1H, 15-H_ax_); 1.61 (m, 1H, 15-H_eq_); 1.49–1.55 (m, 2H, 20-CH_2_CH_2_Ph); 0.96 (s, 3H, 20-CH_3_); 0.70 (dd, ^2^*J*_8α,8β_ = 12.9 Hz, ^3^*J*_8α,7β_ = 8.4 Hz, 1H, 8α-H). ^13^C-NMR δ = 152.4 (C4); 144.7 (Tos-C4); 144.1 (TrC1); 143.2 (C3); 137.2 (Ph-C1); 135.6 (C12); 133.4 (C19); 132.9 (Tos-C1); 130.2 (C18); 129.9 (Tos-C3,5); 129.4 (oCTr); 128.4 (Ph-C3,5); 128.3 (Ph-C2,6); 128.1 (Tos-C2,6); 127.4 (*m*CTr); 127.3 (*p*CTr); 125.5 (C11); 125.2 (Ph-C4); 122.8 (C1); 118.4 (C2); 97.7 (C5); 91.5 (TrCO); 83.4 (C6); 74.3 (C20); 69.7 (6-*O*-CH_2_CH_2_OTos); 65.2 (6-*O*-CH_2_CH_2_OTos); 59.6 (C9); 47.4 (C7); 46.8 (C13); 45.2 (C16); 43.4 (NCH_3_); 42.9 (20-CH_2_CH_2_Ph); 42.7 (C14); 33.5 (C15); 30.7 (C8); 29.1 (20-CH_2_CH_2_Ph); 23.1 (20-CH_3_); 22.3 (C10); 21.7 (Tos-CH_3_). ESI-MS *m/z*: 901 [M+1]^+^ C_57_H_57_NO_7_S (900.13).

*(5R,6R,7R,9R,13S,14R,20R)-(5α,7α)-4,5-Epoxy-6-O-(2-*tert*-butyldiphenylsilyloxyethyl)-α,17-dimethyl-α-(2-phenylethyl)-3-triphenylmethoxy-6,14-ethenomorphinan-7-methanol* (**6**, TBDPSOE-TDPEO). Dry sodium hydride (240 mg, 10 mmol) was suspended in anhydrous *N*,*N*-dimethylformamide (5 mL) under an argon atmosphere. A solution of TDPEO [7] (**1**, 700 mg, 1 mmol) in dry *N*,*N*-dimethylformamide (6 mL) was added and the mixture stirred for 15 min at 0 °C. (2-Bromo-ethoxy) *tert*-butyldiphenylsilane (**15a**, 850 mg, 2.33 mmol) was then added and the reaction mixture left stirring at room temperature overnight. The product mixture was poured into water (50 mL) and the suspension was extracted with dichloromethane (5 × 70 mL). The combined extracts were dried (Na_2_SO_4_) and the solvent was evaporated under vacuum. The crude product was purified by flash chromatography on silica gel [150 g; eluent: hexane-ethyl acetate 7:3 (v/v)]. Yield: 800 mg (81%) yellowish solid; mp. 85–87 °C. R_f_ [A] = 0.91; R_f_ [B] = 0.90; R_f_ [C] = 0.30; R_f_ [D] = 0.63. ^1^H-NMR (CDCl_3_) δ = 7.34–7.72 (m, 10H, (CH_3_)_3_CSi(Ph)_2_); 7.34–7.38 (m, 6H, Tr(*o*)); 7.12–7.27 (m, 5H, 20-CH_2_CH_2_Ph); 7.07–7.11 (m, 9H, Tr(*m,p*)); 6.28 (d, ^2^*J*_2,1_ = 8.2 Hz, 1H, 2-H); 6.11 (d, ^2^*J*_1,2_ = 8.2 Hz, 1H, 1-H); 5.85 (d, ^2^*J*_18,19_ = 8.9 Hz, 1H, 18-H); 5.29 (d, ^2^*J*_19,18_ = 8.9 Hz, 1H, 19-H); 5.03 (br s, 1H, 20-OH); 4.15 (d, ^4^*J*_5β,18_ = 0.9 Hz, 1H, 5β-H); 3.85–3.96 (m, 2H, CH_2_CH_2_OTBDPS); 3.59–3.80 (m, 2H, CH_2_CH_2_OTBDPS); 3.04 (d, ^2^*J*_10β,10α_ = 18.8 Hz, 1H, 10β-H); 2.97 (d, ^3^*J*_9α,10α_ = 6.4 Hz, 1H, 9α-H); 2.81 (m, 1H, 8β-H); 2.73–2.78 (m, 2H, 20-CH_2_CH_2_Ph); 2.38 (dd, ^2^*J*_16eq,16ax _ = 12.0 Hz, ^3^*J*_16eq,15ax_ = 5.1 Hz, 1H, 16-H_eq_); 2.26 (s, 3H, NCH_3_); 2.21 (m, 1H, 16-H_ax_); 2.17 (m, 1H, 10α-H); 1.94 (app t, ^3^*J*_7β,8α_ = 8.3 Hz, 1H, 7β-H); 1.75 (td, ^2^*J*_15ax,15eq_ = 13.7 Hz, ^3^*J*_15ax,16ax_ = 12.8 Hz, ^3^*J*_15ax,16eq_ = 5.6 Hz, 1H, 15-H_ax_); 1.67 (m, 1H, 15-H_eq_); 1.52–1.56 (m, 2H, 20-CH_2_CH_2_Ph); 1.09 (s, 3H, 20-CH_3_); 1.04 (s, 9H, (CH_3_)_3_CSi(Ph)_2_); 0.75 (dd, ^2^*J*_8α,8β_ = 12.9 Hz, ^3^*J*_8α,7β_ = 8.3 Hz, 1H, 8α-H). ^13^C-NMR δ = 152.6 (C4); 144.1 (TrC1); 143.4 (C3); 137.2 (Ph-C1); 135.6 and 135.8 (TBDPS-Ph-C2,6); 134.9 (C12); 133.6 (C19); 133.3 and 133.5 (TBDPS-Ph-C1); 130.2 (C18); 129.6 and 129.6 (TBDPS-Ph-C4); 129.4 (oCTr); 128.5 (Ph-C3,5); 128.2 (Ph-C2,6); 127.7 and 127.7 (TBDPS-Ph-C3,5); 127.2 (mCTr); 127.1 (pCTr); 126.3 (C11); 125.4 (Ph-C4); 122.8 (C1); 118.1 (C2); 97.7 (C5); 91.5 (TrCO); 83.8 (C6); 74.4 (C20); 68.9 (6-*O*-CH_2_CH_2_OTBDPS); 64.1 (6-*O*-CH_2_CH_2_OTBDPS); 59.8 (C9); 47.3 (C7); 46.9 (C13); 45.3 (C16); 43.4 (NCH_3_); 42.9 (20-CH_2_CH_2_Ph); 42.7 (C14); 33.5 (C15); 30.6 (C8); 29.1 (20-CH_2_CH_2_Ph); 26.8 ((CH_3_)_3_CSi(Ph)_2_); 23.4 (20-CH_3_); 22.3 (C10); 19.1 ((CH_3_)_3_CSi(Ph)_2_). ESI-MS *m/z*: 984 [M]^+^ C_66_H_69_NO_5_Si (984.34).

*(5R,6R,7R,9R,13S,14R,20R)-(5α,7α)-4,5-Epoxy-α,17-dimethyl-α-(2-phenylethyl)-3-triphenylmethoxy-6,14-ethenomorphinan-[6,20][1,4]-dioxepane* (**7**, 6,20-ethylendioxy-TDPEO). A solution of TDPEO [7] (**1**, 700 mg, 1 mmol) in *N*,*N*-dimethylformamide (10 mL) was added dropwise to a suspension of dry sodium hydride (240 mg, 10 mmol) and *N*,*N*-dimethylformamide (5 mL) at 0 °C under argon atmosphere. Ethylene glycol ditosylate (370 mg, 1 mmol) dissolved in *N*,*N*-dimethylformamide (10 mL) was added dropwise and the mixture stirred for 24 h at ambient temperature. The solution was poured into ice-water (50 g) and the suspension was extracted with chloroform (4 × 30 mL). The chloroform solution was dried (Na_2_SO_4_) and concentrated under reduced pressure to yield a semisolid, which was purified by chromatography on silica gel [180 g, eluent system: hexane-ethyl acetate 8:2 to 1:1 (v/v)]. Yield: 176 mg (24%). mp. 97–99 °C. R_f_ [A] = 0.86; R_f_ [B] = 0.85; R_f_ [C] = 0.38; R_f_ [D] = 0.58. ^1^H-NMR (CDCl_3_) δ = 7.39–7.42 (m, 6H, Tr(*o*)); 7.20–7.26 (m, 9H, Tr(*m*,*p*)); 7.10–7.19 (m, 5H, 20-CH_2_CH_2_Ph); 6.16 (d, ^2^*J*_2,1_ = 8.2 Hz, 1H, 2-H); 5.99 (d, ^2^*J*_1,2_ = 8.2 Hz, 1H, 1-H); 5.48 (d, ^2^*J*_18,19_ = 8.4 Hz, 1H, 18-H); 5.16 (d, ^2^*J*_19,18_ = 8.4 Hz, 1H, 19-H); 4.33 (d, ^4^*J*_5β,18_ = 1.0 Hz, 1H, 5β-H); 3.91–4.02 (m, 2H, 6-*O*-CH_2_CH_2_O); 3.64–3.73 (m, 2H, 6-*O*-CH_2_CH_2_O); 3.00 (d, ^2^*J*_10β,10α_ = 18.6 Hz, 1H, 10β-H); 2.93 (d, ^3^*J*_9α,10α_ = 6.5 Hz, 1H, 9α-H); 2.80 (m, 1H, 8β-H); 2.60–2.74 (m, 2H, 20-CH_2_CH_2_Ph); 2.37 (dd, ^2^*J*_16eq,16ax_ = 12.2 Hz, ^3^*J*_16eq,15ax_ = 4.9 Hz, 1H, 16-H_eq_); 2.23 (s, 3H, NCH_3_); 2.20 (m, 1H, 16-H_ax_); 2.14 (dd, ^2^*J*_10α,10β_ = 18.6 Hz, ^3^*J*_10α,9α_ = 6.5 Hz, 1H, 10α-H); 2.02 (app t, ^3^*J*_7β,8α_ = 8.9 Hz, 1H, 7β-H); 1.75 (m, 1H, 15-H_ax_); 1.72 (m, 1H, 15-H_eq_); 1.52–1.64 (m, 2H, 20-CH_2_CH_2_Ph); 1.12 (s, 3H, 20-CH_3_); 0.84 (dd, ^2^*J*_8α,8β_ = 13.4 Hz, ^3^*J*_8α,7β_ = 8.9 Hz, 1H, 8α-H). ^13^C-NMR δ = 152.2 (C4); 144.3 (TrC1); 143.1 (C3); 137.7 (Ph-C1); 134.3 (C12); 133.7 (C19); 129.4 (C18); 129.2 (*o*CTr); 128.5 (Ph-C3,5); 128.3 (Ph-C2,6); 127.9 (C11); 127.4 (*m*CTr); 126.9 (*p*CTr); 125.5 (Ph-C4); 121.8 (C1); 117.6 (C2); 91.0 (TrCO); 90.8 (C5); 81.9 (C6); 79.4 (C20); 66.0 (6-*O*-CH_2_CH_2_O); 65.1 (6-*O*-CH_2_CH_2_O); 59.9 (C9); 49.3 (C7); 46.5 (C13); 45.4 (C16); 43.5 (NCH_3_); 43.4 (20-CH_2_CH_2_Ph); 43.2 (C14); 34.2 (C15); 32.4 (C8); 29.4 (20-CH_2_CH_2_Ph); 22.3 (20-CH_3_); 16.2 (C10). ESI-MS *m/z*: 728 [M+1]^+^ C_50_H_49_NO_4_ (727.93).

*(5R,6R,7R,9R,13S,14R,20R)-(5α,7α)-4,5-Epoxy-6-hydroxy-3-methoxy-α,17-dimethyl-α-(2-phenylethyl)-6,14-ethenomorphinan-7-methanol (**9**, DPET). 6-*O*-Desmethyl-phenylethyl thevinol (**9**, DPET) was prepared from 20*R*-phenylethyl thevinol* (**8**, 2.14 g, 4.38 mmol) according to the literature method of Luthra *et al.* [13]. The crude product was purified by column chromatography on silica gel [250 g, eluent system: ethyl acetate-hexane 1:1 (v/v)]. Yield: 1.89 g (91%) mp. 69–70 °C R_f_ [chloroform-methanol 9:1] = 0.78; R_f_ [C] 0.10; R_f_ [D] = 0.23. ^1^H-NMR (CDCl_3_) δ = 7.14–7.29 (m, 5H, 20-CH_2_CH_2_Ph); 6.62 (d, ^2^*J*_2,1_ = 8.1 Hz, 1H, 2-H); 6.52 (d, ^2^*J*_1,2_ = 8.1 Hz, 1H, 1-H); 5.68 (d, ^2^*J*_18,19_ = 8.6 Hz, 1H, 18-H); 5.32 (d, ^2^*J*_19,18_ = 8.6 Hz, 1H, 19-H); 4.43 (br s, 1H, 20-OH); 4.36 (d, ^4^*J*_5β,18_ = 1.1 Hz, 1H, 5β-H); 3.82 (s, 3H, 3-OCH_3_); 3.56 (br s, 1H, 6-OH); 3.21 (d, ^2^*J*_10β,10α_ = 18.5 Hz, 1H, 10β-H); 3.11 (d, ^3^*J*_9α,10α_ = 6.5 Hz, 1H, 9α-H); 2.85 (dd, ^2^*J*_8β,8α_ = 12.8 Hz, ^3^*J*_8β,7α_ = 9.3 Hz, 1H, 8β-H); 2.75–2.81 (m, 2H, 20-CH_2_CH_2_Ph); 2.48 (dd, ^2^*J*_16eq,16ax_ = 12.0 Hz, ^3^*J*_16eq,15ax_ = 4.8 Hz, 1H, 16-H_eq_); 2.38 (m, 1H, 16-H_ax_); 2.36 (m, 1H, 10α-H); 2.34 (s, 3H, NCH_3_); 2.03 (t, ^3^*J*_7β,8α_ = 8.1 Hz, 1H, 7β-H); 1.93 (td, ^2^*J*_15ax,15eq_ = 13.1 Hz, ^3^*J*_15ax,16ax_ = 12.8 Hz, ^3^*J*_15ax,16eq_ = 5.5 Hz, 1H, 15-H_ax_); 1.83 (dd, ^2^*J*_15eq,15ax_ = 13.1 Hz, ^3^*J*_15eq,16ax_ = 2.7 Hz, 1H, 15-H_eq_); 1.58–1.76 (m, 2H, 20-CH_2_CH_2_Ph); 1.11 (s, 3H, 20-CH_3_); 0.84 (dd, ^2^*J*_8α,8β_ = 12.8 Hz, ^3^*J*_8α,7β_ = 8.1 Hz, 1H, 8α-H). ^13^C-NMR δ = 148.0 (C4); 143.0 (C3); 141.7 (Ph-C1); 134.7 (C12); 134.6 (C19); 130.0 (C18); 128.4 (Ph-C3,5); 128.4 (C11); 128.3 (Ph-C2,6); 125.6 (Ph-C4); 119.6 (C1); 112.9 (C2); 98.0 (C5); 78.9 (C6); 75.1 (C20); 59.9 (C9); 56.4 (3-OCH_3_); 46.7 (C7); 46.7 (C13); 45.4 (C16); 43.5 (NCH_3_); 43.2 (20-CH_2_CH_2_Ph); 42.9 (C14); 33.6 (C15); 30.5 (C8); 29.1 (20-CH_2_CH_2_Ph); 23.8 (20-CH_3_); 21.0 (C10). ESI-MS *m/z*: 474 [M+1]^+^ C_30_H_35_NO_4_ (473.60).

*(5R,6R,7R,9R,13S,14R,20R)-(5α,7α)-4,5-Epoxy-6-(2-fluoroethoxy)-3-methoxy-α,17-dimethyl-α-(2-phenylethyl)-6,14-ethenomorphinan-7-methanol* (**10**, FE-DPET). A solution of DPET (**9**, 630 mg, 1.33 mmol) in *N*,*N*-dimethylformamide (8 mL) was added dropwise over 5 min to a suspension of dry sodium hydride (320 mg, 13.3 mmol) in *N*,*N*-dimethylformamide (6 mL) under argon at 0 °C. Thereafter, 1-Bromo-2-fluoroethane (395 mg, 230 μL, 3.1 mmol) was added at 0 °C, the mixture allowed to warm to room temperature and was stirred for 48 h. The mixture was slowly added into water (50 mL). After extraction (dichloromethane, 4 × 70 mL), drying (Na_2_SO_4_), filtration and solvent removal the crude product was obtained as a yellowish oil, which was purified by column chromatography [silica gel, 150 g, eluent: hexane-ethyl acetate 7:3 (v/v)]. Yield 360 mg (52%) yellow oil; ^1^H-NMR (CDCl_3_) δ = 7.15–7.29 (m, 5H, PhCH_2_CH_2_); 6.62 (d, ^2^*J*_2,1_ = 8.2 Hz, 1H, 2-H); 6.52 (d, ^2^*J*_1,2_ = 8.2 Hz, 1H, 1-H); 5.94 (d, ^2^*J*_18,19_ = 8.9 Hz, 1H, 18-H); 5.45 (d, ^2^*J*_19,18_ = 8.9 Hz, 1H, 19-H); 4.85 (br s, 1H, 20-OH); 4.53–4.71 (m, 2H, 6-*O*-CH_2_CH_2_F); 4.57 (s, 1H, 5β-H); 4.19–4.43 (m, 2H, 6-*O*-CH_2_CH_2_F); 3.81 (s, 3H, 3-OCH_3_); 3.20 (d, ^2^*J*_10β,10α_ = 18.4 Hz, 1H, 10β-H); 3.10 (d, ^3^*J*_9α,10α_ = 6.5 Hz, 1H, 9α-H); 2.88 (dd, 1H, ^2^*J*_8β,8α_ = 12.8 Hz, ^3^*J*_8β,7β_ = 8.8 Hz, 8β-H); 2.74–2.84 (m, 2H, PhCH_2_CH_2_); 2.48 (dd, ^2^*J*_16eq,16ax_ = 12.1 Hz, ^3^*J*_16eq,15ax_ = 4.9 Hz, 1H, 16-H_eq_); 2.39 (m, 1H, 16-H_ax_); 2.36 (m, 1H, 10α-H); 2.34 (s, 3H, NCH_3_); 2.08 (app t, 1H, ^3^*J*_7β,8α_ = 8.7 Hz, 7β-H); 1.93 (td, ^2^*J*_15ax,15eq_ = 14.0 Hz, ^3^*J*_15ax,16ax_ = 13.0 Hz, ^3^*J*_15ax,16eq_ = 5.6 Hz, 1H, 15H_ax_); 1.83 (dd, 1H, ^2^*J*_15eq,16ax_ = 13.3 Hz, ^3^*J*_15eq,16ax_ = 2.6 Hz, 15-H_eq_); 1.57–1.75 (m, 2H, PhCH_2_CH_2_); 1.09 (s, 3H, 20-CH_3_); 0.84 (dd, ^2^*J*_8α,8β_ = 12.8 Hz, ^3^*J*_8α,7β_ = 8.4 Hz, 1H, 8α-H). ^13^C-NMR (CDCl_3_) δ = 147.9 (C4); 143.2 (C3); 141.7 (Ph-C1); 135.7 (C12); 134.2 (C19); 128.5 and 128.3 (Ph-C3,5 and Ph-2,6); 128.4 (C18); 125.5 (Ph-C4); 125.4 (C11); 119.4 (C1); 113.5 (C2); 98.6 (C5); 84.4 (C6); 83.1 (d, *J* = 169.6 Hz, 6-*O*-CH_2_CH_2_F); 74.6 (C20); 66.8 (d, *J* = 18.3 Hz, 6-*O*-CH_2_CH_2_F); 59.8 (C9); 56.6 (3-OCH_3_); 47.6 (C7); 47.1 (C13); 45.4 (C16); 43.5 (NCH_3_); 43.0 (C14); 42.9 (PhCH_2_CH_2_); 33.6 (C15); 30.7 (C8); 29.1 (PhCH_2_CH_2_); 23.2 (20-CH_3_); 22.2 (C10). ^19^F-NMR δ = −224.7 ESI-MS *m/z*: 520 [M+1]^+^ C_32_H_38_FNO_4_ (519.65).

*(5R,6R,7R,9R,13S,14R,20R)-(5αα)-4,5-Epoxy-6-ethoxy-3-methoxy-α,17-dimethyl-α-(2-phenylethyl)-6,14-ethenomorphinan-7-methanol* (**11**, E-DPET). 1M L-Selectride (0.7 mL, 0.7 mmol, 3 eq) in THF was added to a cooled solution of FE-DPET (**10**, 119 mg, 0.23 mmol) in THF (5 mL) at 0 °C. The mixture was refluxed for 3 h. The resulting solution was cooled and poored into water (20 mL) and extracted with dichloromethane-methanol 9:1 (v/v, 3 × 30 mL). The combined organic layer was washed with water (20 mL) and dried (Na_2_SO_4_). The solvent was evaporated under vacuum and the residue was chromatographed [silica gel, 50 g, eluent: ethyl acetate-hexane 1:1 (v/v)] to yield 104 mg (90%) of **11** as a pale, yellowish oil. ^1^H-NMR (CDCl_3_): δ = 7.13–7.27 (m, 5H, 20-CH_2_CH_2_Ph); 6.60 (d, ^2^*J*_2,1_ = 8.0 Hz, 1H, 2-H); 6.48 (d, ^2^*J*_1,2_ = 8.0 Hz, 1H, 1-H); 5.93 (d, ^2^*J*_18,19_ = 9.0 Hz, 1H, 18-H); 5.41 (d, ^2^*J*_19,18_ = 9.0 Hz, 1H, 19-H); 5.92 (br s, 1H, 20-OH); 4.52 (d, ^4^*J*_5β,18_ = 0.5 Hz, 1H, 5β-H); 4.22 (m, 1H, one of 6-*O*-CH_2_CH_3_); 3.95 (m, 1H, other 6-OCH_2_CH_3_); 3.80 (s, 3H, 3-OCH_3_); 3.18 (d, ^2^*J*_10β,10α_ = 18.5 Hz, 1H, 10β-H); 3.07 (d, ^3^*J*_9α,10α_ = 6.0 Hz, 1H, 9α-H); 2.84 (dd, ^2^*J*_8β,8α_ = 12.9 Hz, ^3^*J*_8β,7α_ = 9.0 Hz, 1H, 8β-H); 2.72–2.80 (m, 2H, 20-CH_2_CH_2_Ph); 2.46 (dd, ^2^*J*_16eq,16ax_ = 12.2 Hz, ^3^*J*_16eq,15ax_ = 4.9 Hz, 1H, 16-H_eq_); 2.37 (m, 1H, 16_ax_-H); 2.34 (m, 1H, 10α-H); 2.32 (s, 3H, NCH_3_); 2.01 (app t, ^3^*J*_7β,8α_ = 9.0 Hz, 1H, 7β-H); 1.91 (td, ^2^*J*_15ax,15eq_ = 13.4 Hz, ^3^*J*_15ax,16ax_ = 12.7 Hz, ^3^*J*_15ax,16eq_ = 5.4 Hz, 1H, 15_ax_-H); 1.80 (dd, ^2^*J*_15eq,15ax_ = 13.2 Hz, ^3^*J*_15eq,16ax_ = 2.6 Hz, 1H, 15_eq_-H); 1.54–1.71 (m, 2H, 20-CH_2_CH_2_Ph); 1.29 (t, *J* = 7 Hz, 3H, 6-OCH_2_CH_3_); 1.05 (s, 3H, 20-CH_3_); 0.79 (dd, ^2^*J*_8α,8β_ = 12.9 Hz, ^3^*J*_8α,7β_ = 8.0 Hz, 1H, 8α-H). ^13^C-NMR (CDCl_3_): δ = 148.0 (C4); 143.2 (C3); 141.7 (Ph-C1); 135.3 (C12); 134.3 (C19); 128.5 (Ph-C3,5); 128.4 (C18); 128.3 (Ph-C2,6); 125.7 (Ph-C4); 125.5 (C11); 119.2 (C1); 113.8 (C2); 99.0 (C5); 83.9 (C6); 74.6 (C20); 63.2 (6-*O*-CH_2_CH_3_); 59.9 (C9); 56.8 (3-OCH_3_); 47.5 (C7); 47.1 (C13); 45.4 (C16); 43.5 (NCH_3_); 43.1 (C14); 42.8 (20-CH_2_CH_2_Ph); 33.6 (C15); 30.5 (C8); 29.1 (20-CH_2_CH_2_Ph); 23.3 (20-CH_3_); 22.2 (C10); 16.5 (6-*O*-CH_2_CH_3_). ESI-MS *m/z*: 502 [M+1]^+^ C_32_H_39_NO_4_ (501.66).

*(5R,6R,7R,9R,13S,14R,20R)-(5α,7α)-4,5-epoxy-3-hydroxy-6-methoxy-α,17-dimethyl-α-(2-phenylethyl)-6,14-ethenomorphinan-7-methanol* (**12**, PEO). 20*R*-Phenylethyl thevinol (**8**, 7 g, 14.35 mmol) was dissolved in tetrahydrofuran (20 mL). 1M L-Selectride (76 mL, 76 mmol, 5.3 eq) was added dropwise at 0 °C, and the solution was then refluxed for 5 h. After cooling to room temperature the mixture was poured into ice-water (150 g), and extracted with dichloromethane-methanol 9:1 (v/v, 4 × 80 mL). The organic phases were combined, dried (Na_2_SO_4_) and concentrated under vacuum to give **12** as a beige solid. The crude product was purified by column chromatography on silica gel as described earlier [7]. The product was found to be identical with PEO (**12**) prepared in our laboratory earlier [7] (physical constants, ^1^H-NMR and ^13^C-NMR spectra). Yield: 4.11 g (60%).

*(2-Bromoethoxy)-tert-butyldiphenylsilane* (**15a**) 2-Bromethanol (**14**, 3.17 g, 1.8 mL, 25 mmol) was dissolved in dry dichloromethane (5 mL). *N*-ethyldiisopropylamine (3.59 g, 4.6 mL, 27.77 mmol) was added and the mixture stirred at room temperature for 15 min. *tert*-Butyldiphenylsilyl chloride (TBDPSCl, 7.71 g, 7.3 mL, 28 mmol) and DMAP (20 mg) were added and the reaction mixture was stirred for 48 h at ambient temperature. The solvent was removed under vacuum and the crude product was purified by flash column chromatography on silica gel [320 g, eluent: hexane-ethyl acetate 20:1 (v/v)]. Yield: 8.76 g (95%) colourless oil. R_f_ [hexane-ethyl acetate 20:1] = 0.76. ^1^H-NMR (CDCl_3_) δ = 7.36–7.67 (m, 10H, (CH_3_)_3_CSi(Ph)_2_); 3.90 (t, *J* = 6.5 Hz, 2H, CH_2_CH_2_OTBDPS); 3.40 (t, *J* = 6.5 Hz, 2H, CH_2_CH_2_Br); 1.05 (s, 9H, (CH_3_)_3_CSi(Ph)_2_). ^13^C-NMR δ = 135.5 (C-2,6); 133.2 (Ar); 129.8 (C4 and C1); 127.7 (C-3,5); 63.9 (CH_2_OTBDPS); 33.1 (BrCH_2_CH_2_); 26.7 ((CH_3_)_3_C); 19.2 (CH_3_)_3_C). C_18_H_23_BrOSi (363.36).

*(2-Bromoethoxy)-tert-butyldimethylsilane* (**15b**) This product was prepared analogously to **15a** from 2-bromethanol (3.17 g, 25 mmol) using *tert*-butyldimethylsilyl chloride (TBDMSCl, 4.23 g, 28 mmol). The crude product was purified by column chromatography [340 g silica gel, eluent: hexane-ethyl acetate 20:1 (v/v)]. Yield: 4.23 g (69%) colourless oil. R_f_ [hexane-ethyl acetate 20:1] = 0.74. ^1^H-NMR (CDCl_3_) δ = 3.89 (t, *J* = 6.7 Hz, 2H, BrCH_2_CH_2_O); 3.39 (t, *J* = 6.7 Hz, 2H, BrCH_2_CH_2_O); 0.90 (s, 9H, C(CH_3_)_3_(CH_3_)Si); 0.09 (s, 6H, C(CH_3_)_3_(CH_3_)_2_Si). ^13^C-NMR δ = 63.5 (BrCH_2_CH_2_O); 33.2 (BrCH_2_CH_2_O); 25.8 (C(CH_3_)_3_(CH_3_)_2_Si); −5.26 (C(CH_3_)_3_(CH_3_)_2_Si). C_8_H_19_BrOSi (239.23).

### 3.3. Radiosynthesis of 6-O-(2-[^18^F]Fluoroethyl)-6-O-desmethylphenylethyl orvinol *([^18^F]FE-PEO, [^18^F] **3**)*

[^18^F]fluoride was produced through the ^18^O(*p*,*n*)^18^F nuclear reaction. The [^18^F]fluoride was obtained in a 34 mM solution of K_2_CO_3_ (0.3 mL) and added to a 2 mL conical vial containing dry MeCN (0.5 mL) and Kryptofix 2.2.2 (15 mg, 39.9 μmol). The solvent was evaporated by heating at reduced pressure. Azeotropic drying was repeated three times with 0.5 mL portions of MeCN. TE-TDPEO (2 mg, 2.2 μmol) was dissolved in anhydrous MeCN (200 µL) and added to the dried [K ⊂ 2.2.2]^+18^F^−^, the vial was then sealed and heated at 90 °C for 10 min. After this, the reactor was cooled to 40 °C prior to the addition of 1 M HCl in EtOH (1 mL). The resulting mixture was stirred at 40 °C for 5 min, neutralised by the addition of a 1 M NH_4_OH solution (1.05 mL) and transferred for preparative HPLC. The preparative HPLC system consisted of a Chromolith^®^ C18 column (ID: 10 mm; length: 100 mm; VWR, Oslo, Norway) eluted with a mobile phase consisting of MeCN: 0.1% ammonium formate (35:65, v/v) at a flow rate of 10 mL/min. In-line HPLC detectors included a UV detector set at 254 nm and a radioactivity detector. The fraction containing the product was collected and diluted 1:1 with water. The mixture was applied to a Sep-Pak C18 solid-phase extraction cartridge (Waters), the cartridge was subsequently washed with 10 mL water. The product was eluted with 0.5 mL ethanol and diluted to the desired concentration (total volume 10 mL) with phosphate buffered saline. The pH of the final solution was between 7 and 8. The isolated product co-eluted with an authentic reference of FE-PEO (**3**) in two different analytical HPLC systems. System 1: Chromolith RP18e (4.6 × 100 mm; acetonitrile /0.1 M ammonium formate (27.5: 72.5 (v/v), 5 mL/min, *k*’ = 6.5. System 2: Nucleosil 100 CN (4.6 × 250 mm; acetonitrile/0.1 M ammonium formate 50:50 (v/v), 2 ml/min, *k* = 5.2. Radiochemical and chemical purities were >99% as determined by analytical HPLC**.**

## 4. Conclusions

The cold reference substance 6-*O*-(2-fluoroethyl)-6-*O*-desmethylphenylethyl orvinol (FE-PEO, **3**) was synthesized via two separate reaction routes. An efficient three step procedure was developed for the preparation of the precursor TE-TDPEO (**5**) starting from 3-*O*-trity-6-*O*-desmethylphenylethyl orvinol (TDPEO, **1**) for the synthesis of ([^18^F]FE-PEO, [^18^F]**3**), a new opioid agonist tracer, by direct nucleophilic radiofluorination and subsequent deprotection. The practical utility of the method is demonstrated by the relevantly high yield and purity of the radiosynthesis of [^18^F]FE-PEO by methodology that is adaptable to production runs on a routine basis. Both compounds, non radioactive-[FE-PEO (**3**)], as well as radioactive [^18^F]FE-PEO ([^18^F]**3**), are now available for further *in vitro* and *in vivo* studies.

## Supplementary Materials

Supplementary materials can be accessed at: http://www.mdpi.com/1420-3049/17/10/11554/s1.
