# Supplementary material for: Design and Synthesis of an 18F-Labeled Version of Phenylethyl Orvinol ([18F]FE-PEO) for PET-Imaging of Opioid Receptors
Source: Molecules. 2012 Sep 28;17(10):11554–69. doi: 10.3390/molecules171011554 (PMC6268392; doi:10.3390/molecules171011554)

**Figure S1.** $^{1}$H-NMR spectrum of FE-TDPEO (**2**) in $CDCl_3$.

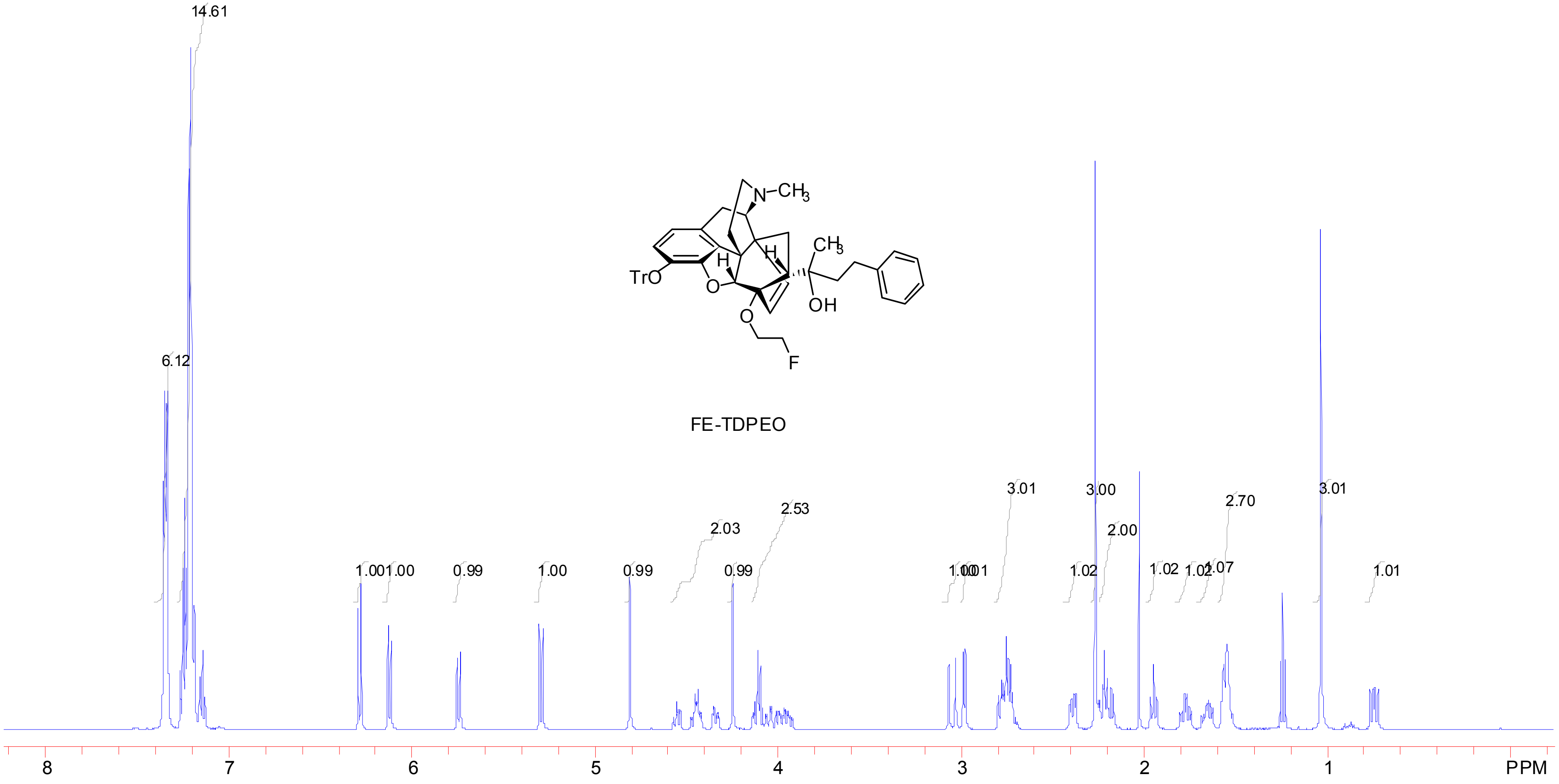

**Figure S2.** $^{13}$C-NMR spectrum of FE-TDPEO (**2**) in $CDCl_3$.

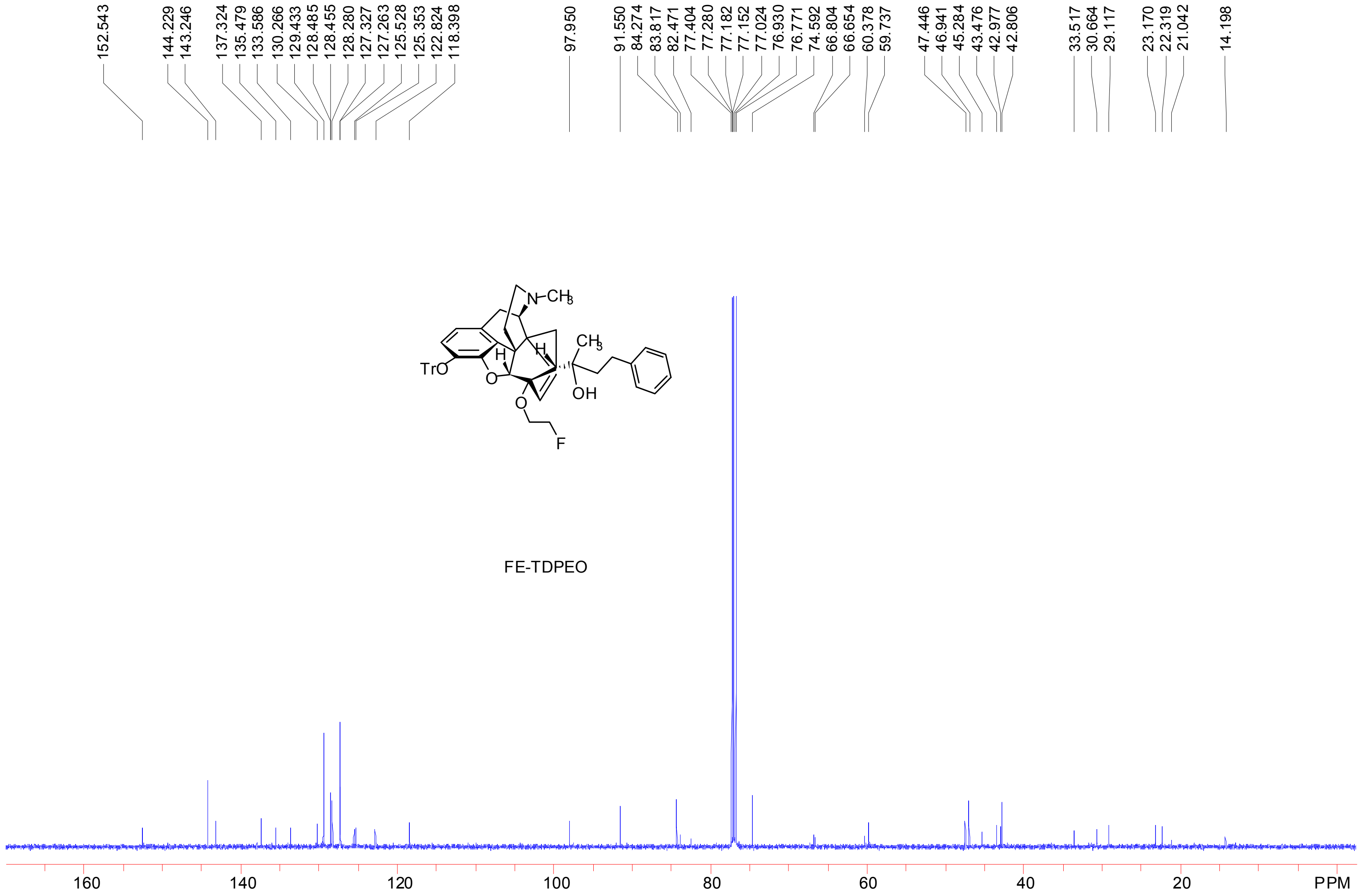

**Figure S3.** $^{19}$F-NMR spectrum of FE-TDPEO (**2**) in $CDCl_3$.

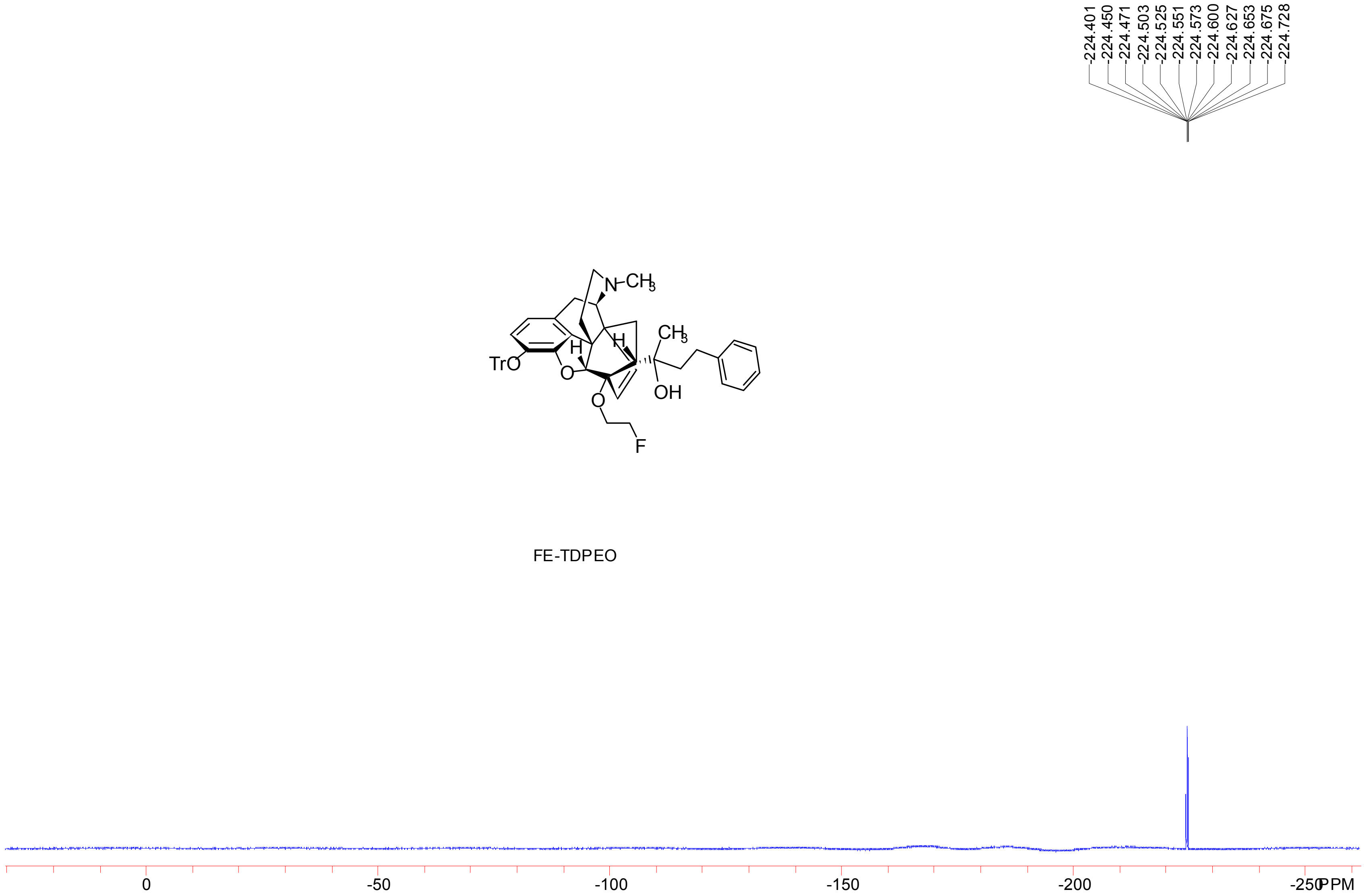

**Figure S4.** ESI-MS of FE-TDPEO (**2**) in $CDCl_3$.

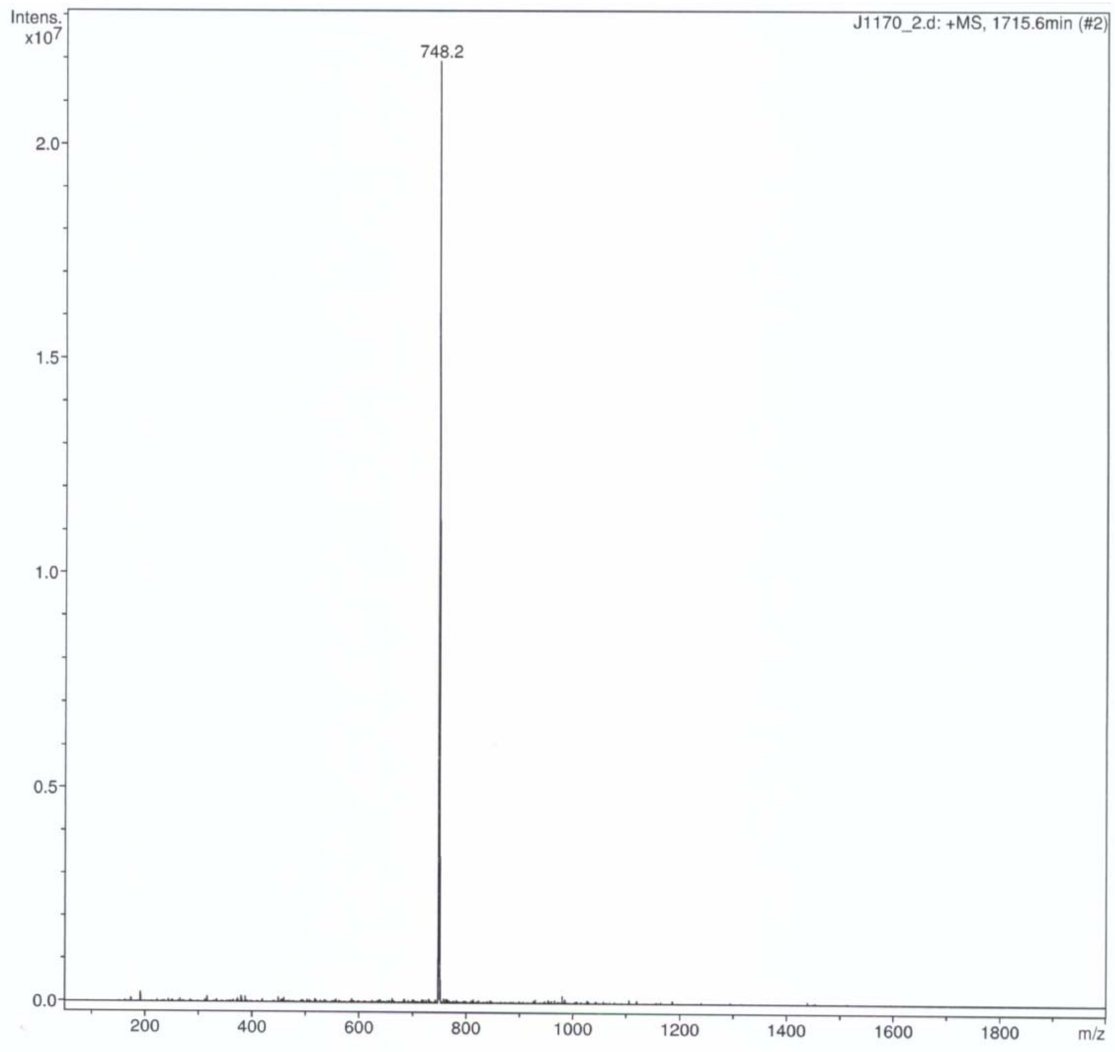

**Figure S5.** $^{1}$H-NMR spectrum of FE-PEO (**3**) in $CDCl_3$.

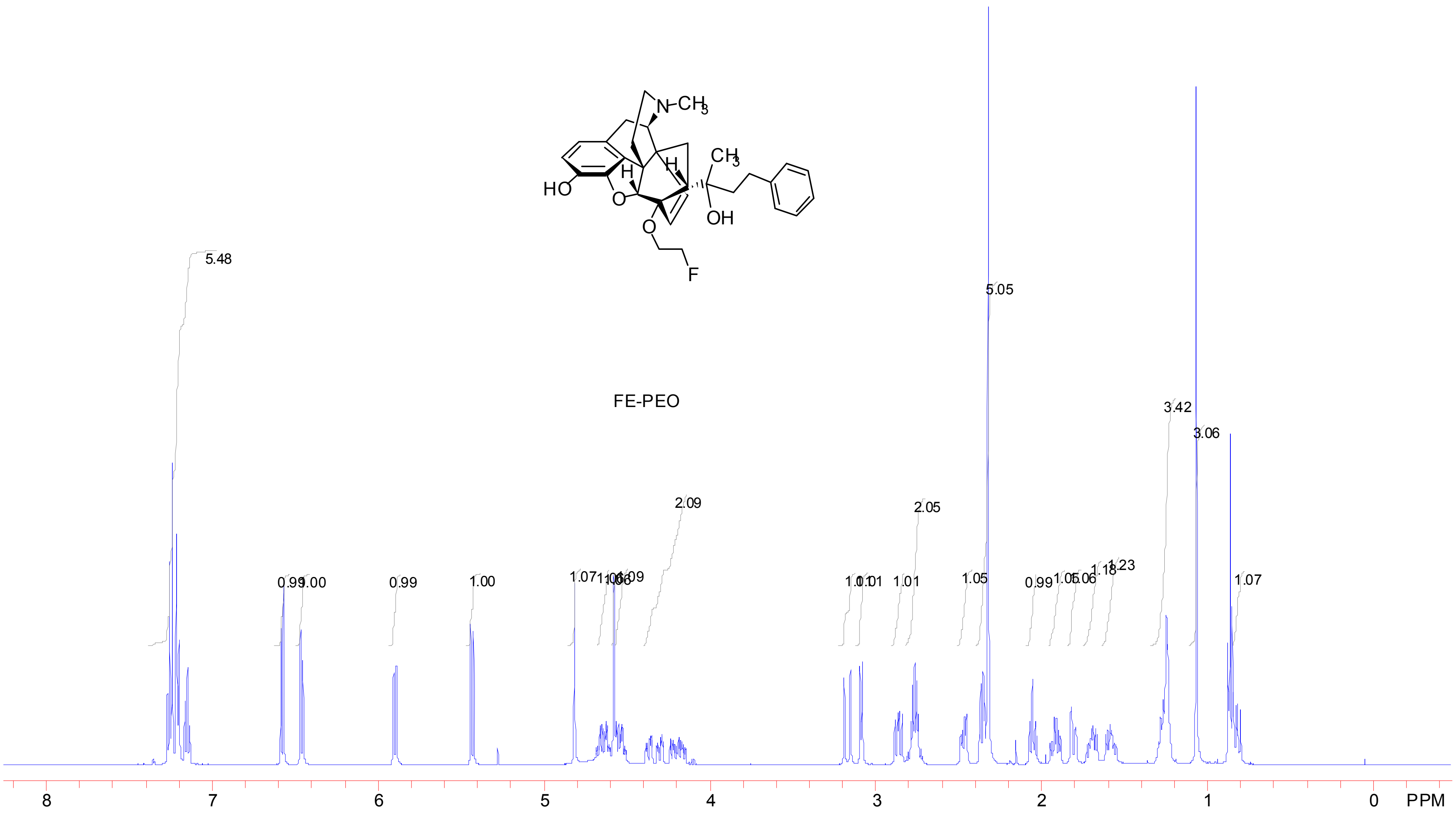

**Figure S6.** $^{13}$C-NMR spectrum of FE-PEO (**3**) in $CDCl_3$.

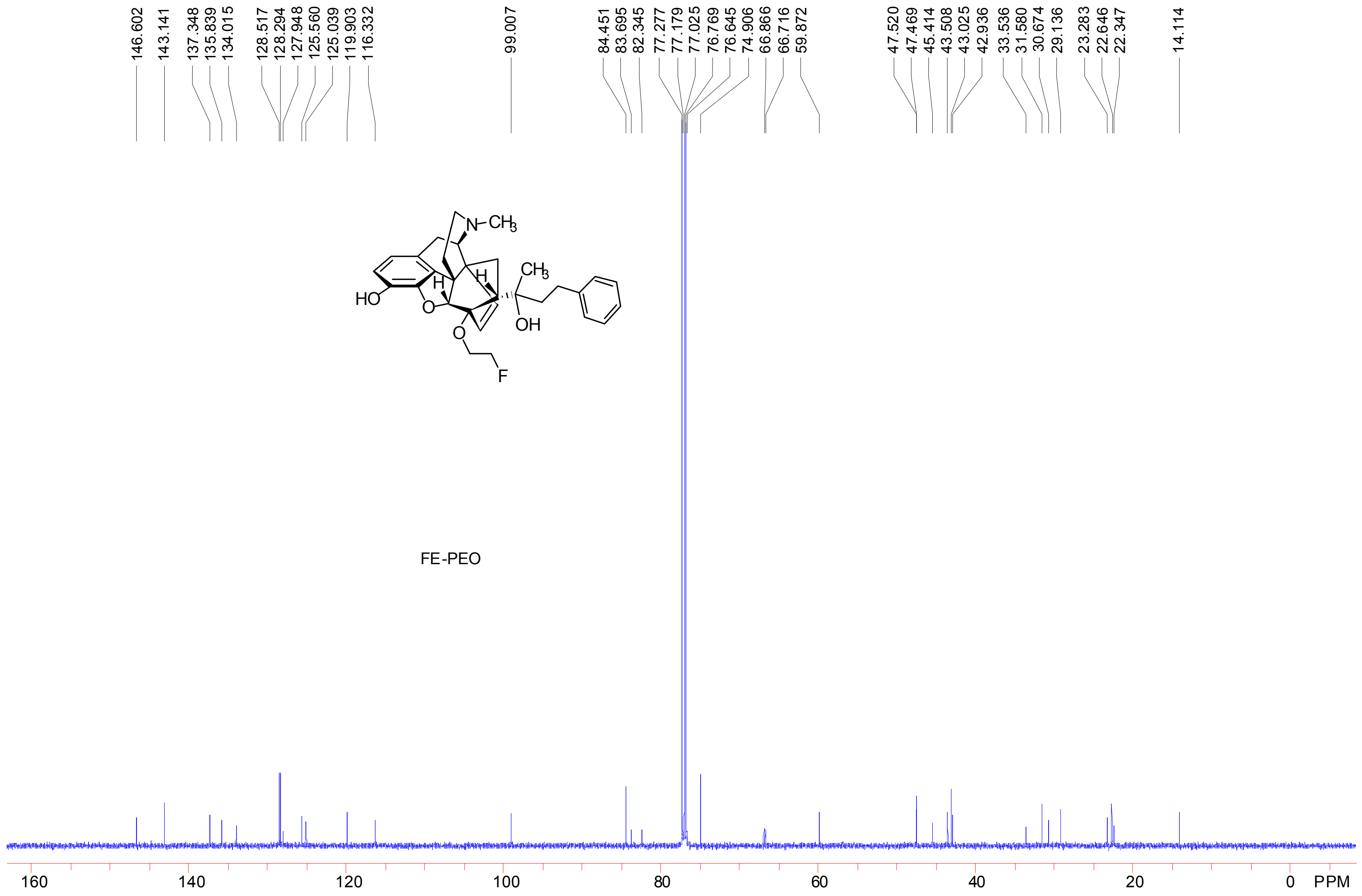

**Figure S7.** $^{19}$F-NMR spectrum of FE-PEO (**3**) in $CDCl_3$.

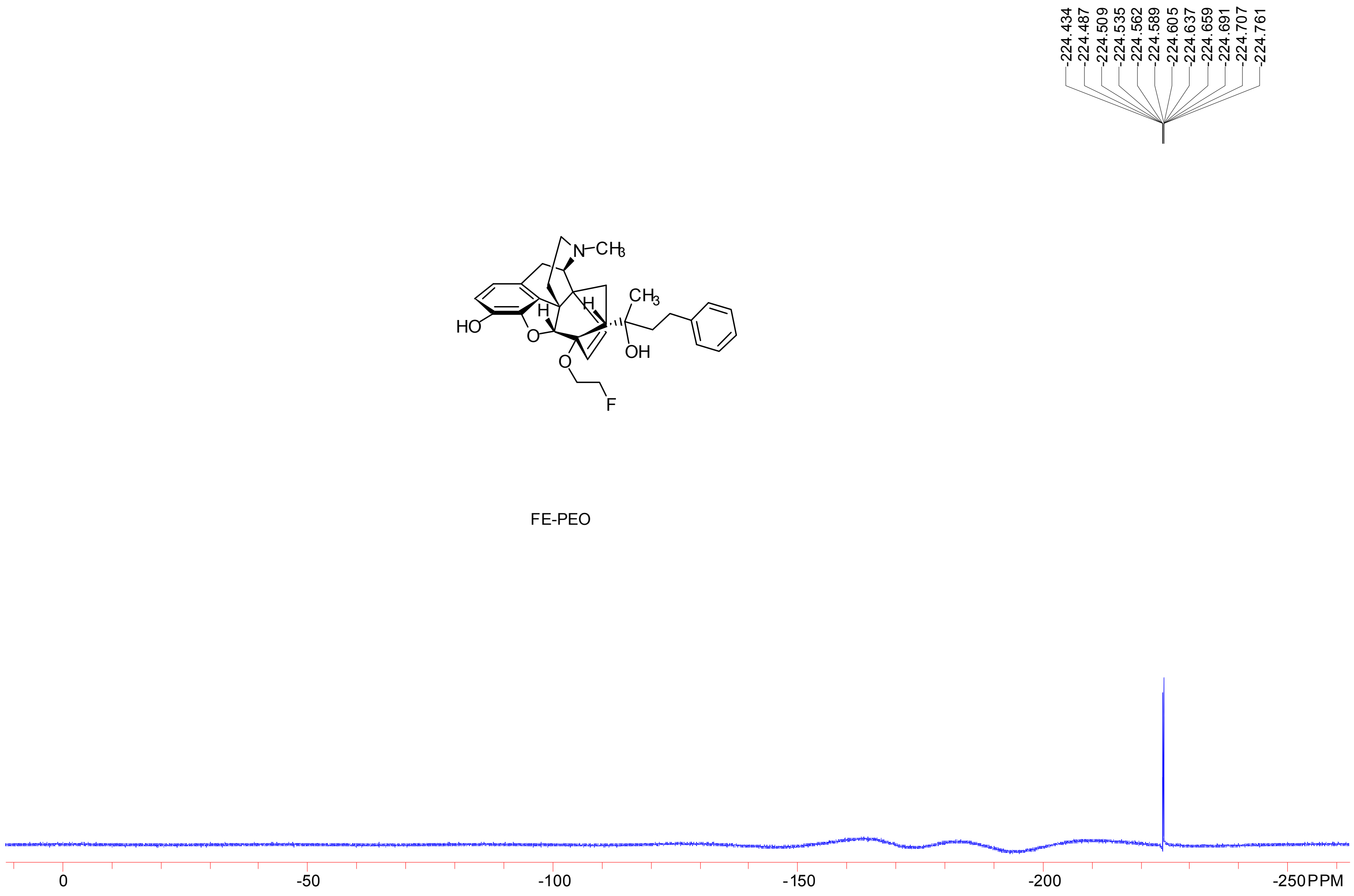

**Figure S8.** ESI-MS of FE-PEO (**3**).

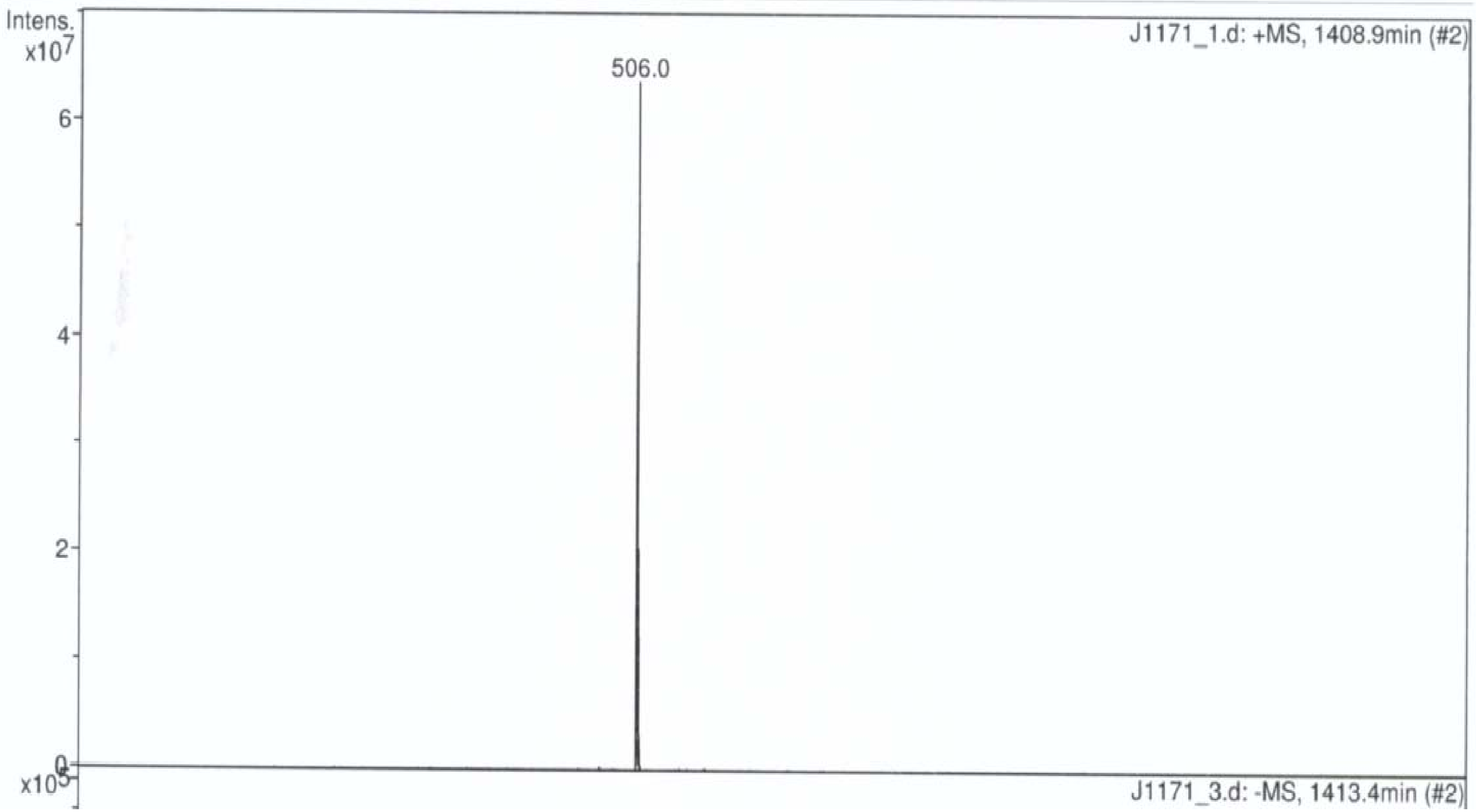

**Figure S9.** $^{1}$H-NMR spectrum of HE-TDPEO (**4**) in $CDCl_3$.

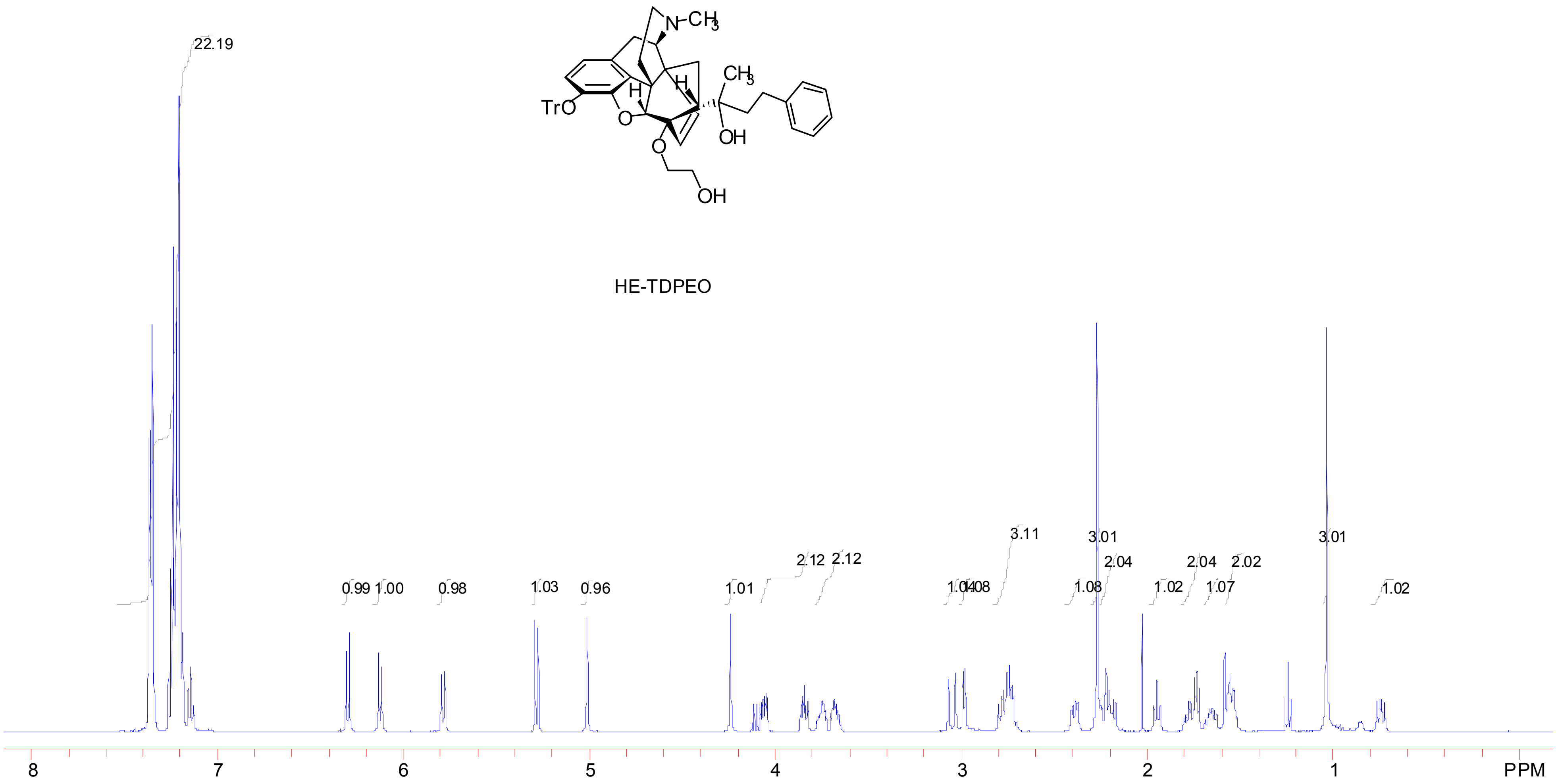

**Figure S10.** $^{13}$C-NMR spectrum of HE-TDPEO (**4**) in $CDCl_3$.

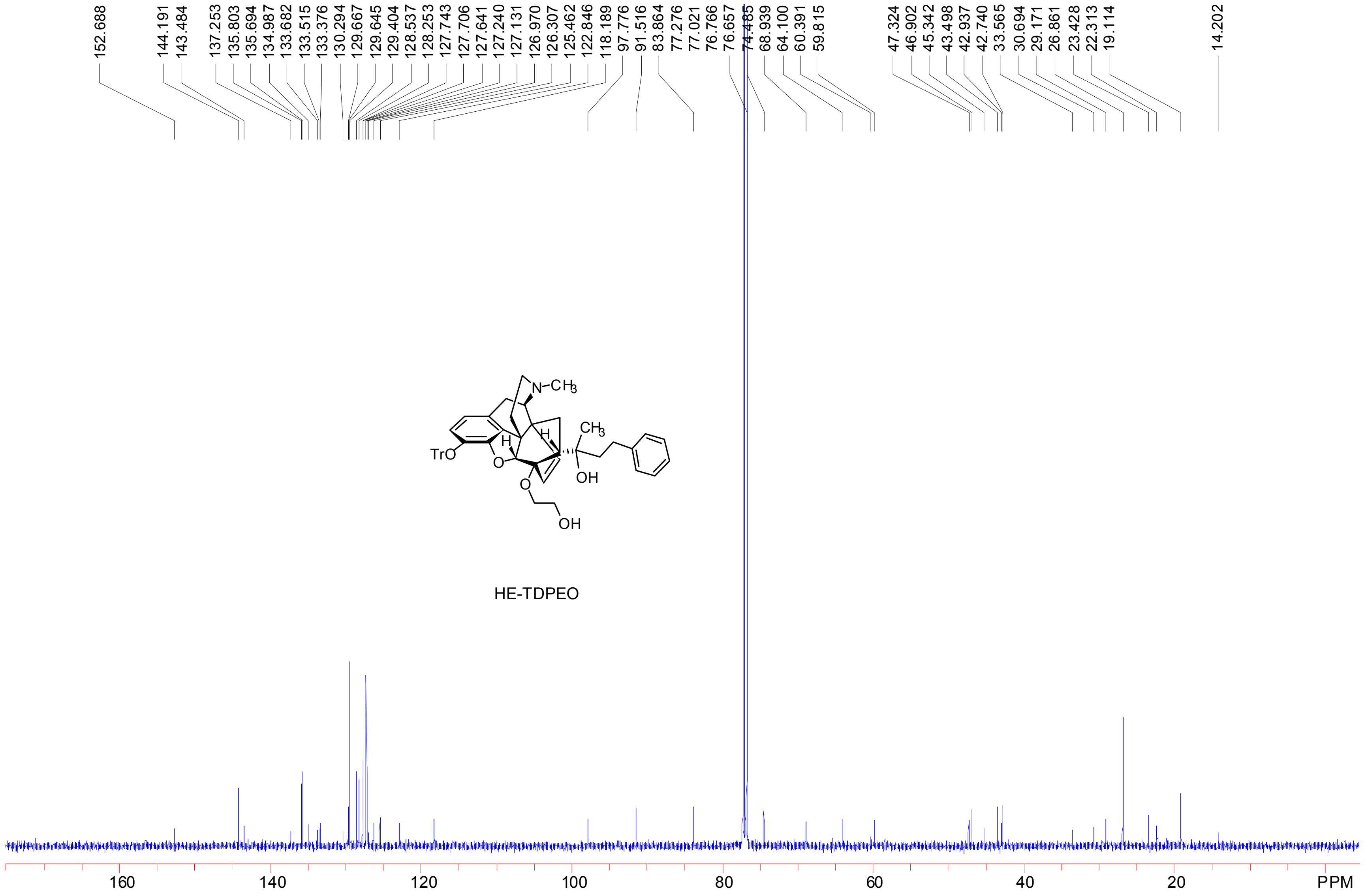

**Figure S11.** ESI-MS of HE-TDPEO (**4**).

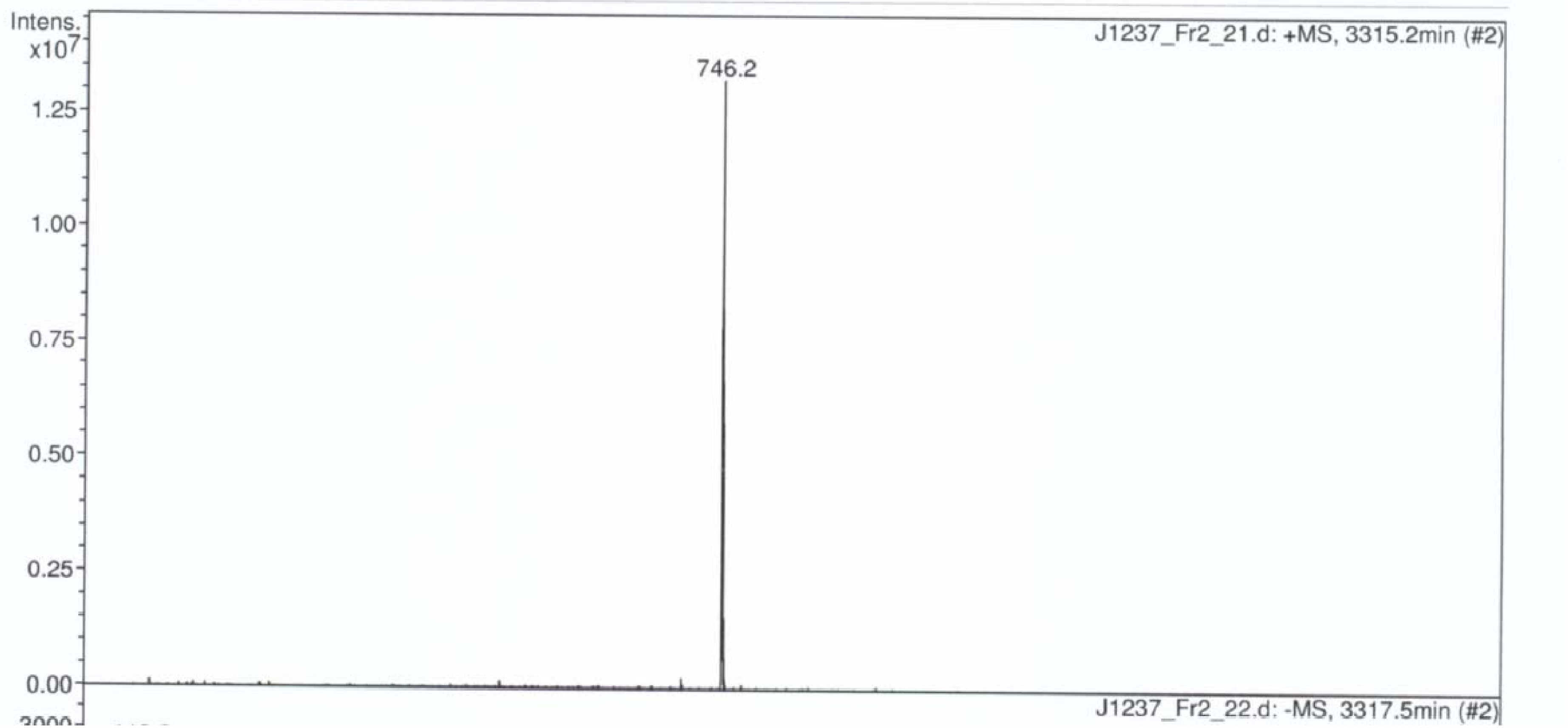

**Figure S12.** $^1$H-NMR spectrum of TE-TDPEO (**5**) in $CDCl_3$.

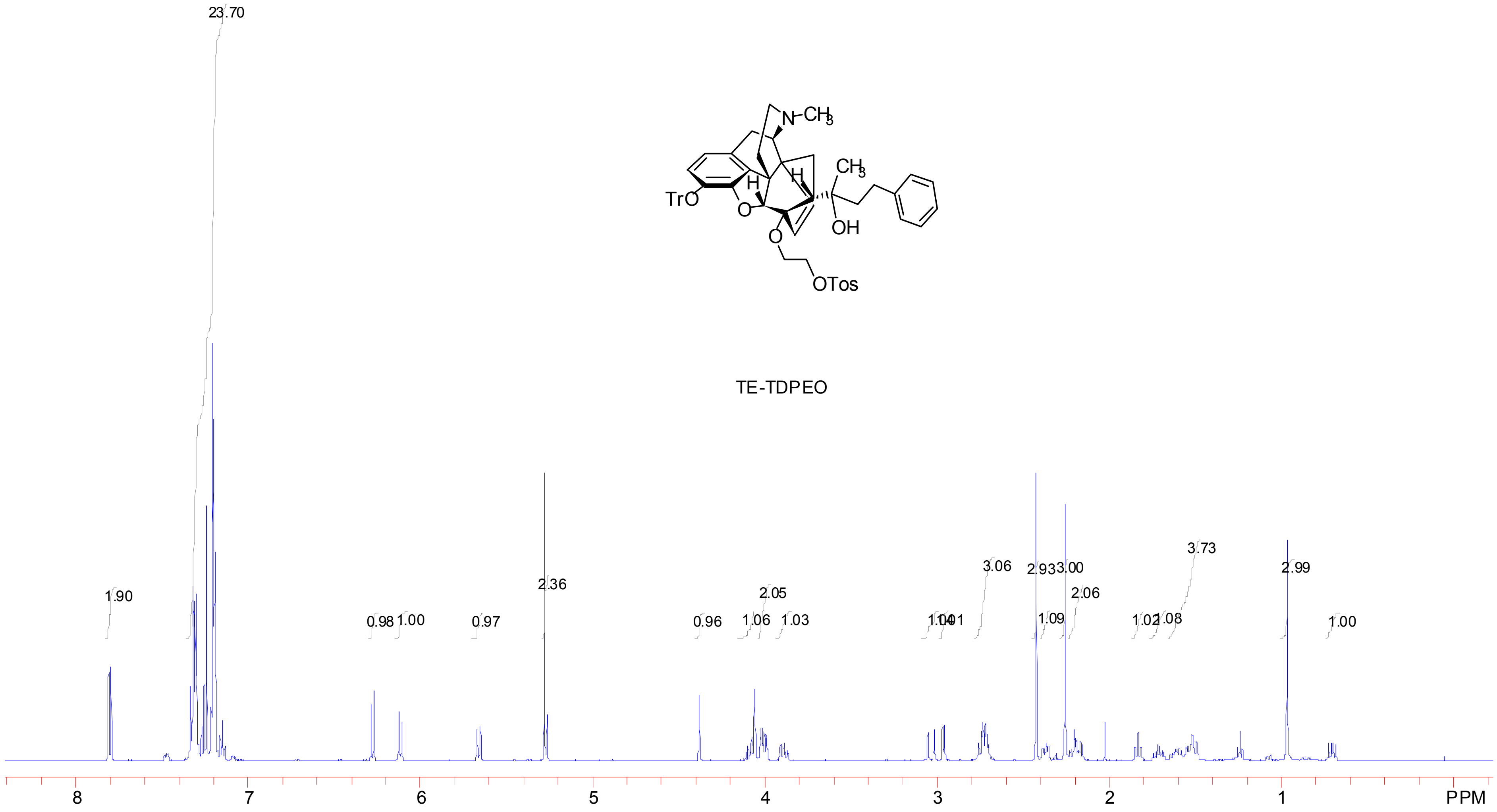

**Figure S13.** $^{13}$C-NMR spectrum of TE-TDPEO (**5**) in $CDCl_3$

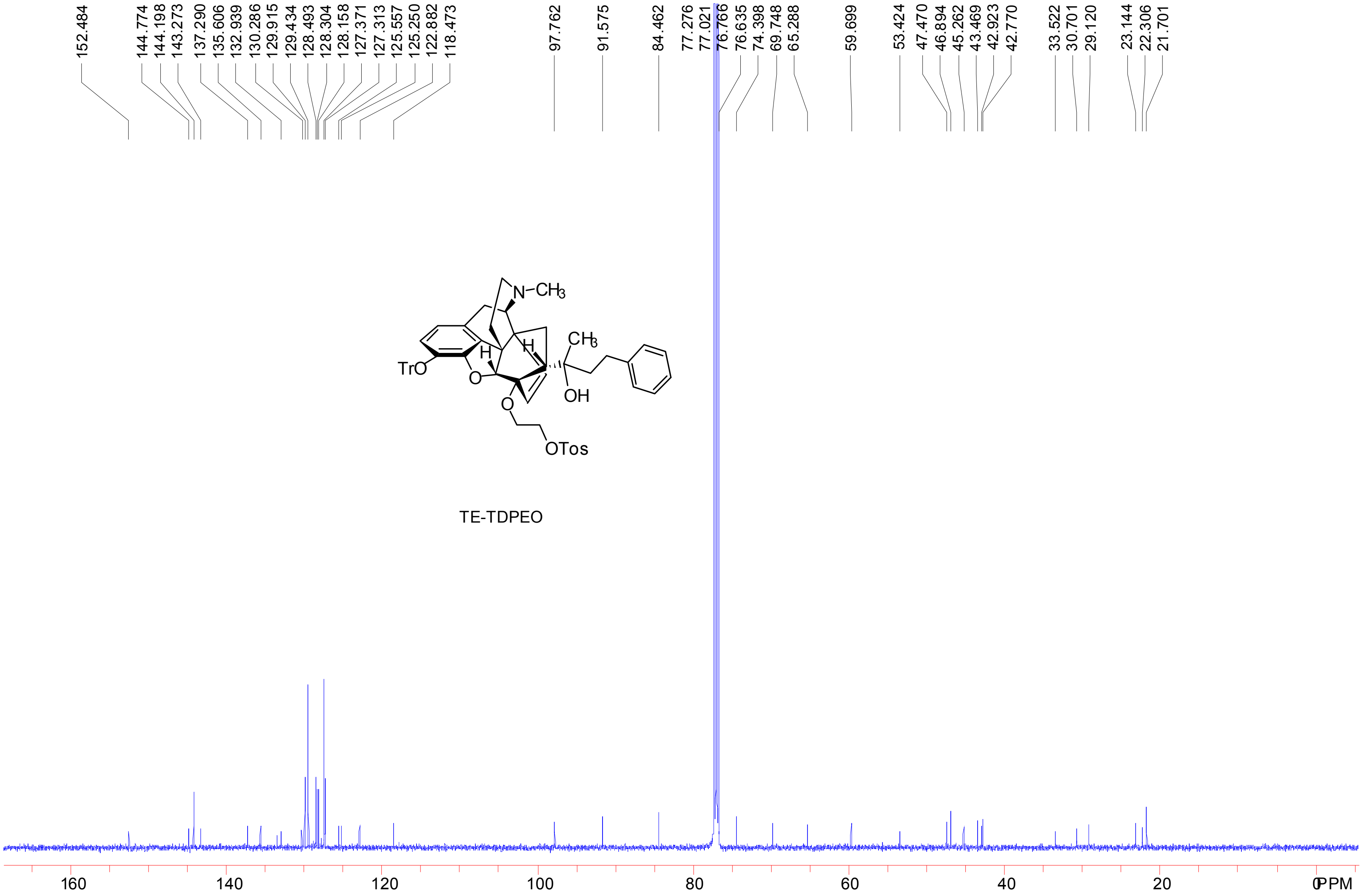

**Figure S14.** ESI-MS of TE-TDPEO (**5**).

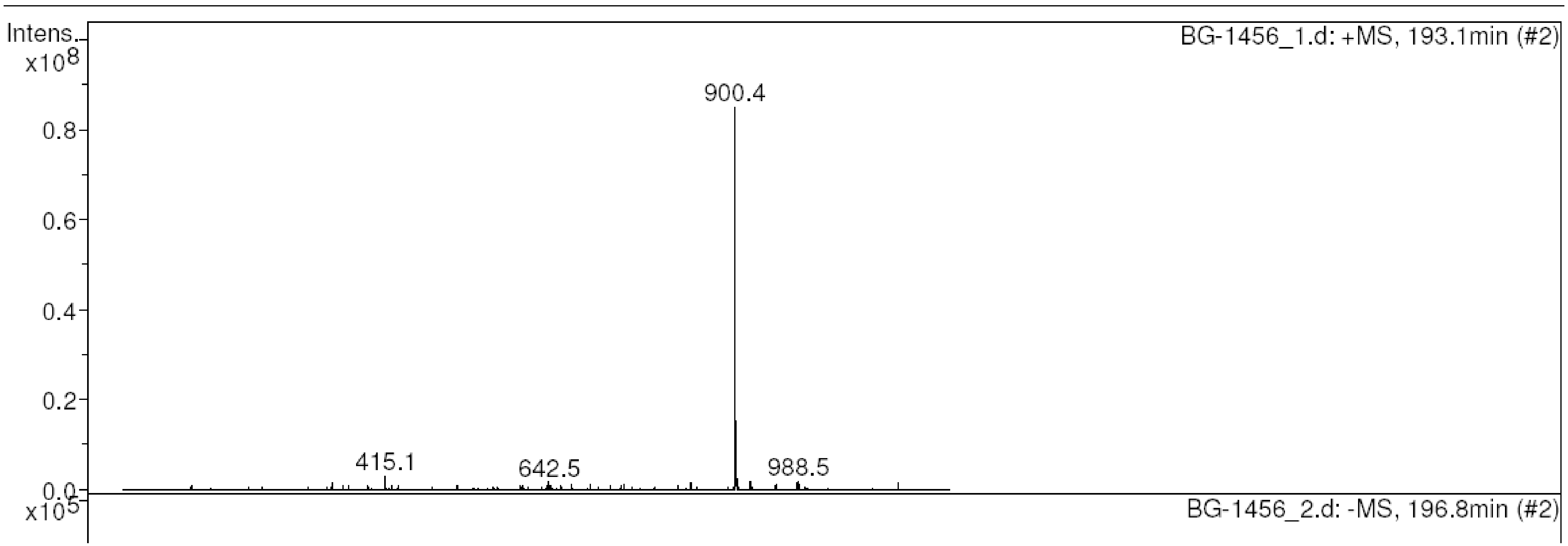

**Figure S15.** $^{1}$H-NMR spectrum of TBDPSOE-TDPEO (**6**) in $CHCl_3$.

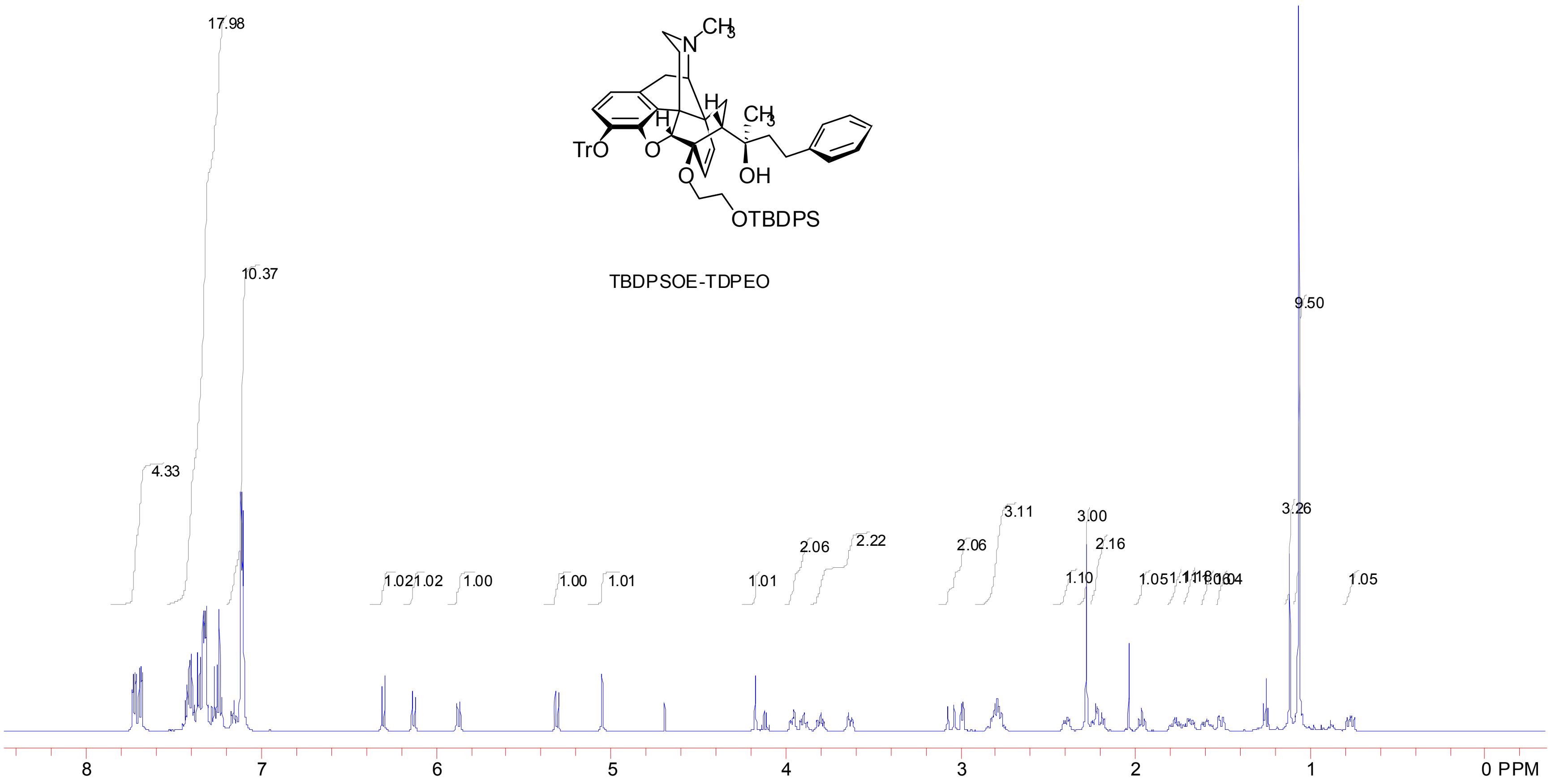

**Figure S16.** $^{13}$C-NMR spectrum of TBDPSOE -TDPEO (**6**) in $CDCl_3$.

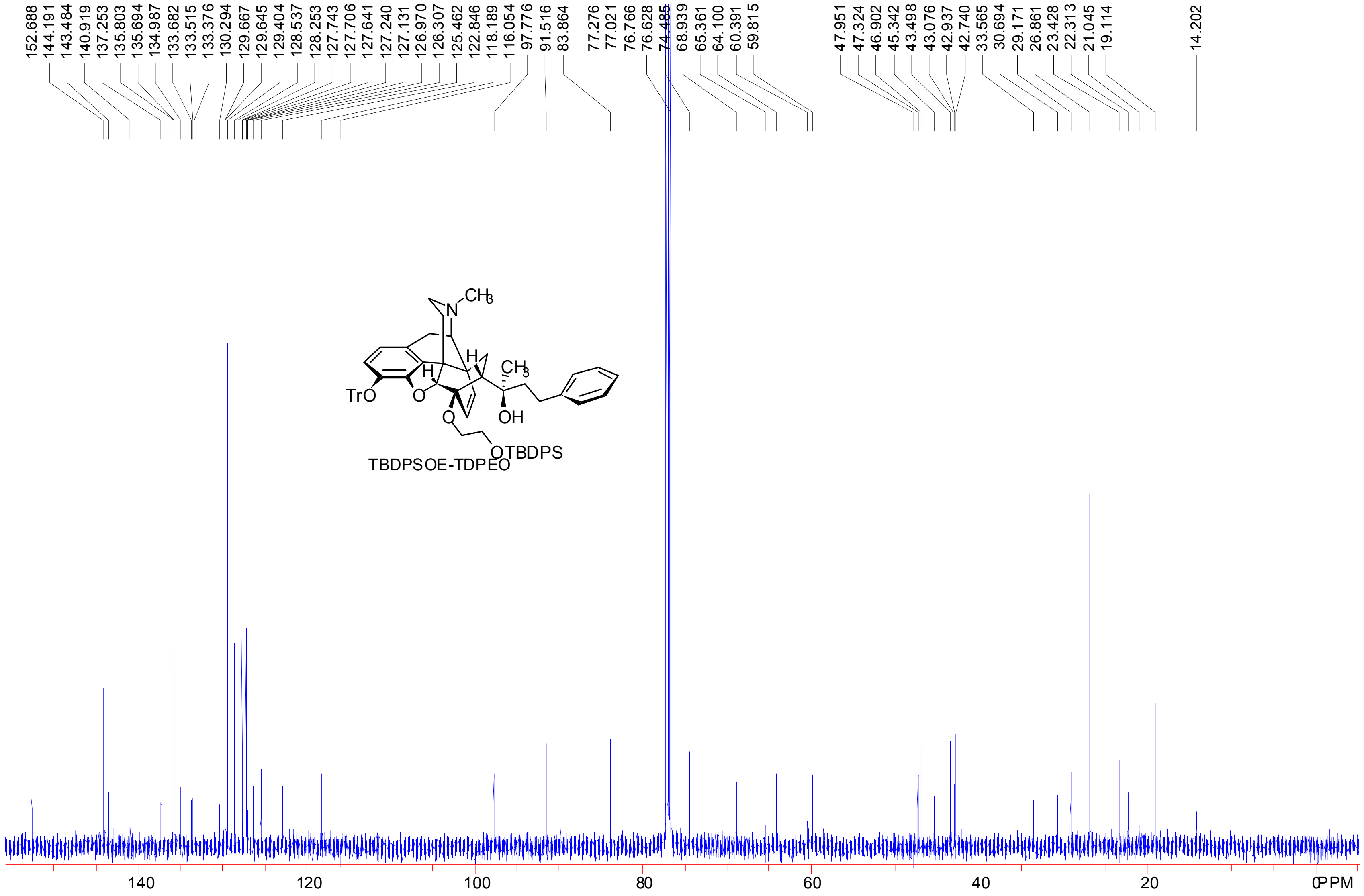

**Figure S17.** ESI-MS of TBDPSOE -TDPEO (**6**).

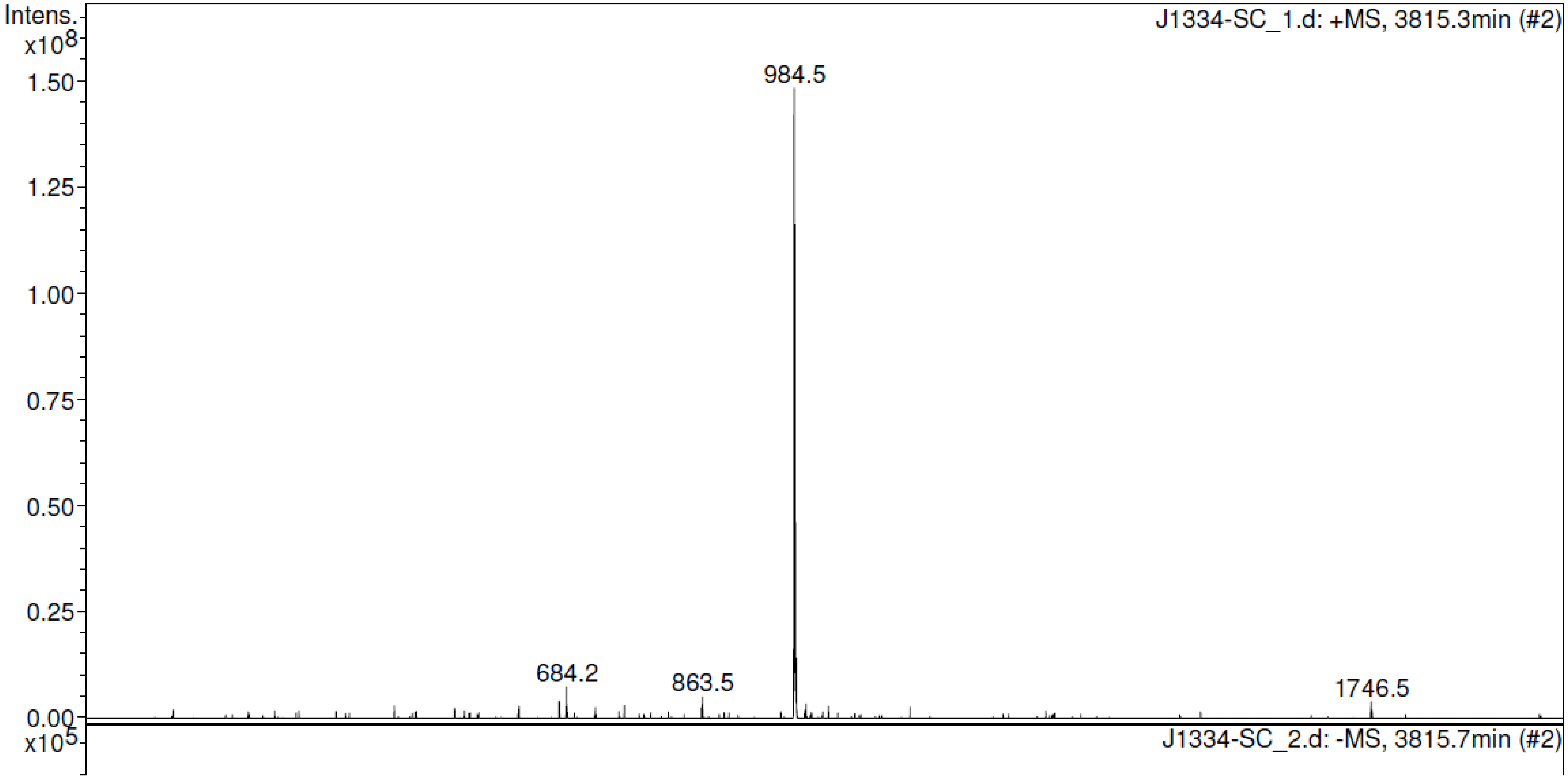

**Figure S18.** $^1$H-NMR spectrum of 6,20-ethylenedioxy-TDPEO (**7**) in $CDCl_3$.

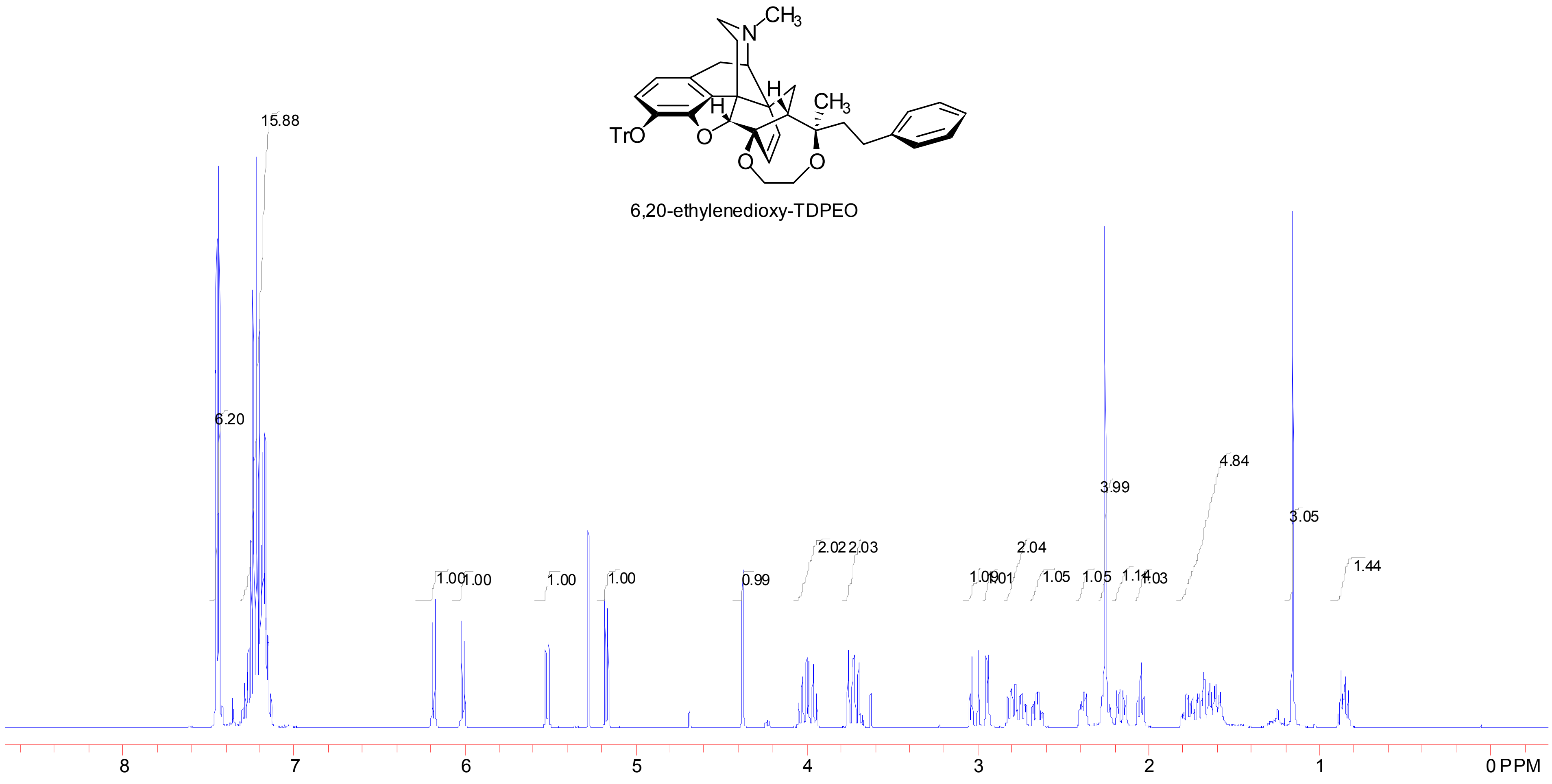

**Figure S19.** $^{13}$C-NMR spectrum of 6,20-ethylenedioxy -TDPEO (**7**) in $CDCl_3$.

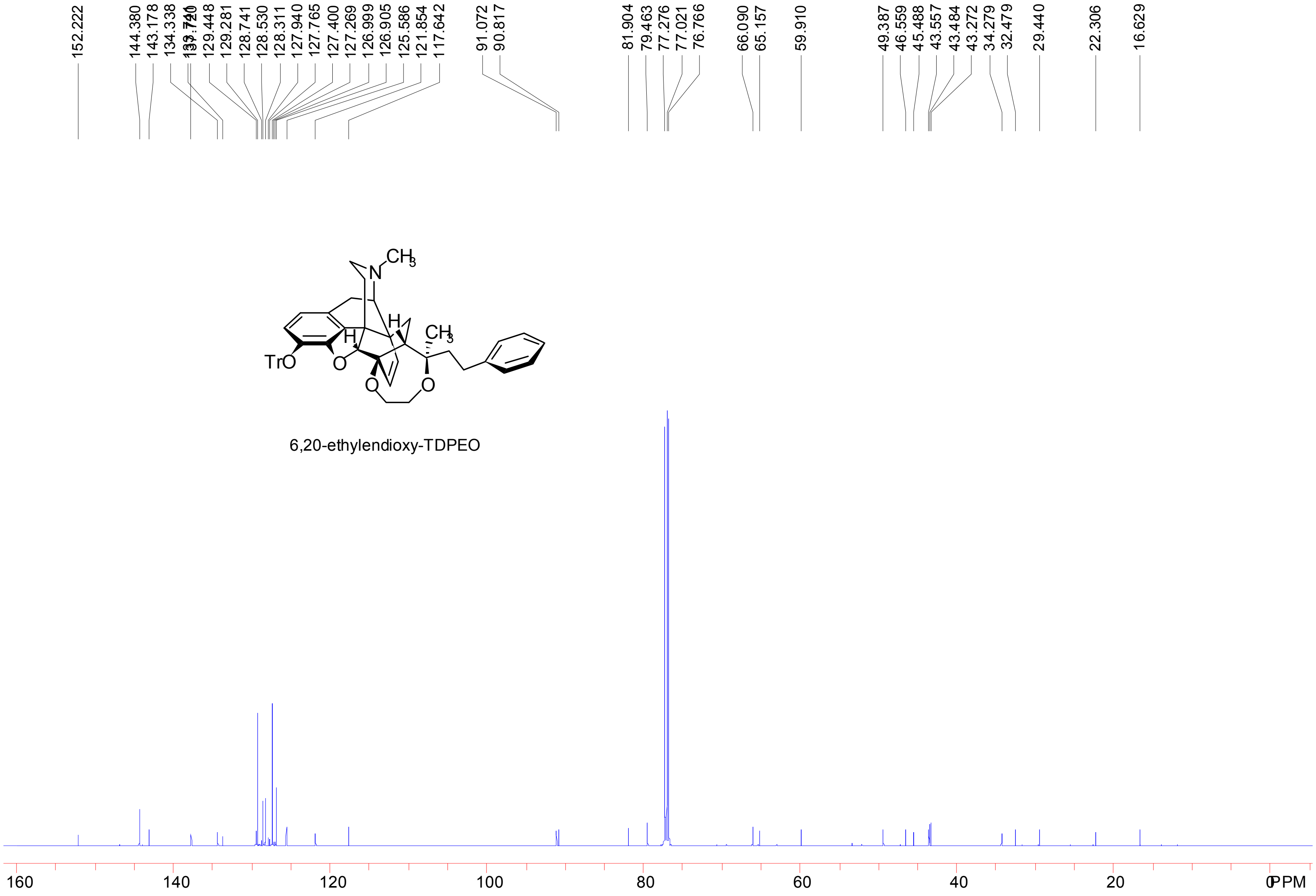

**Figure S20.** ESI-MS of 6,20-ethylenedioxy -TDPEO (**7**).

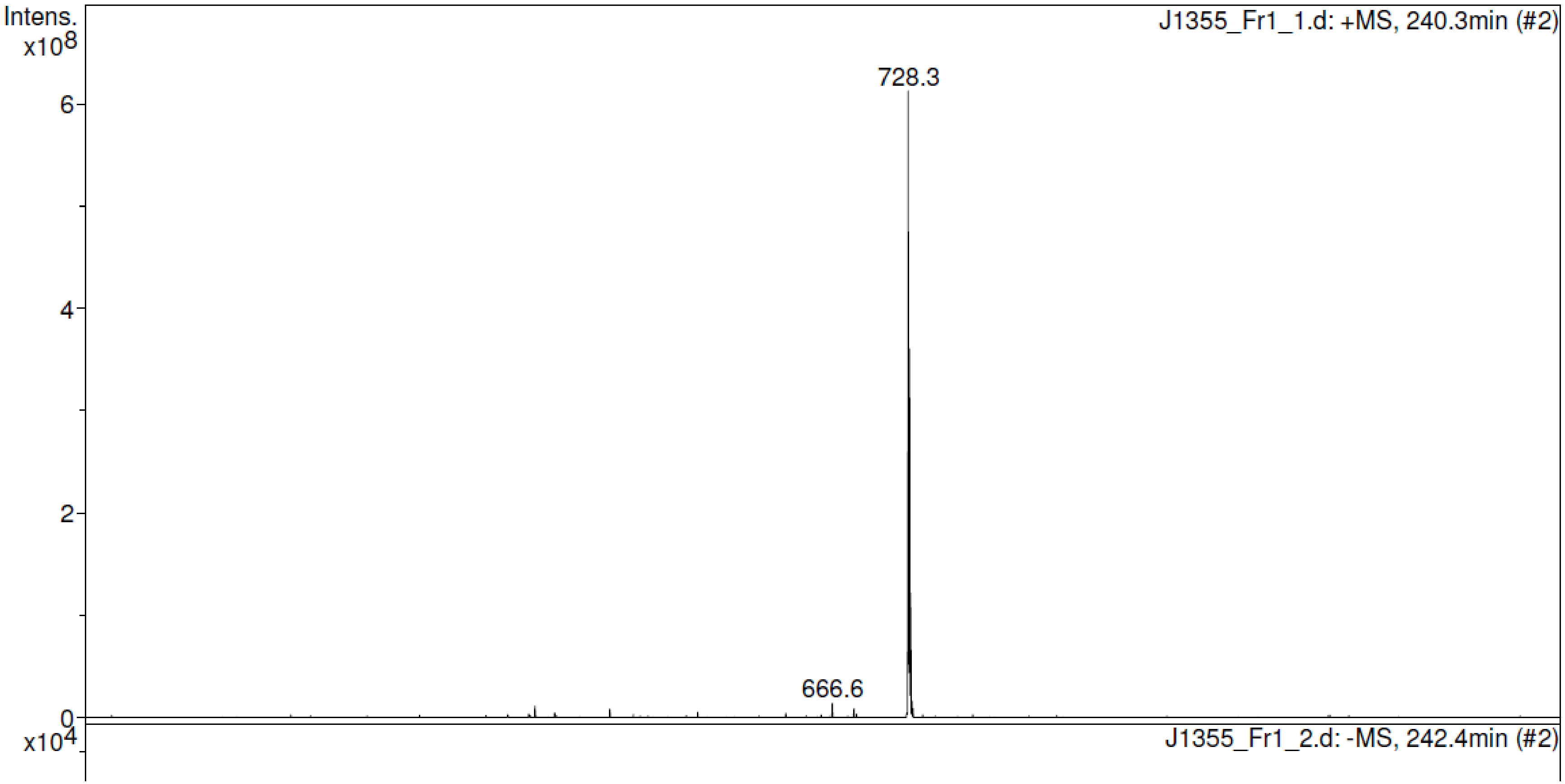

**Figure S21.** $^{1}$H-NMR spectrum of DPET (**9**) in $CDCl_3$.

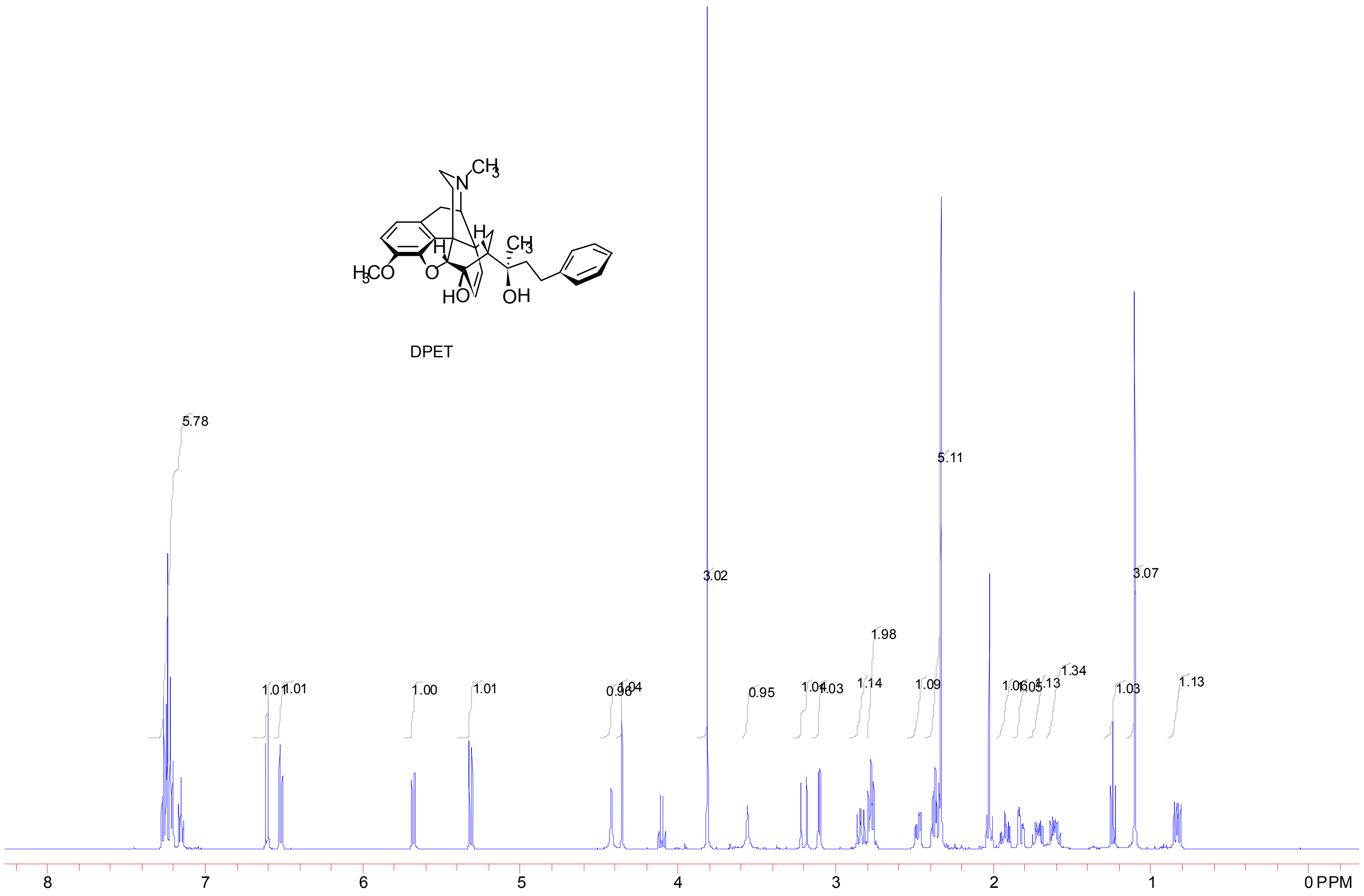

**Figure S22.** $^{13}$C-NMR spectrum of DPET (**9**) in $CDCl_3$.

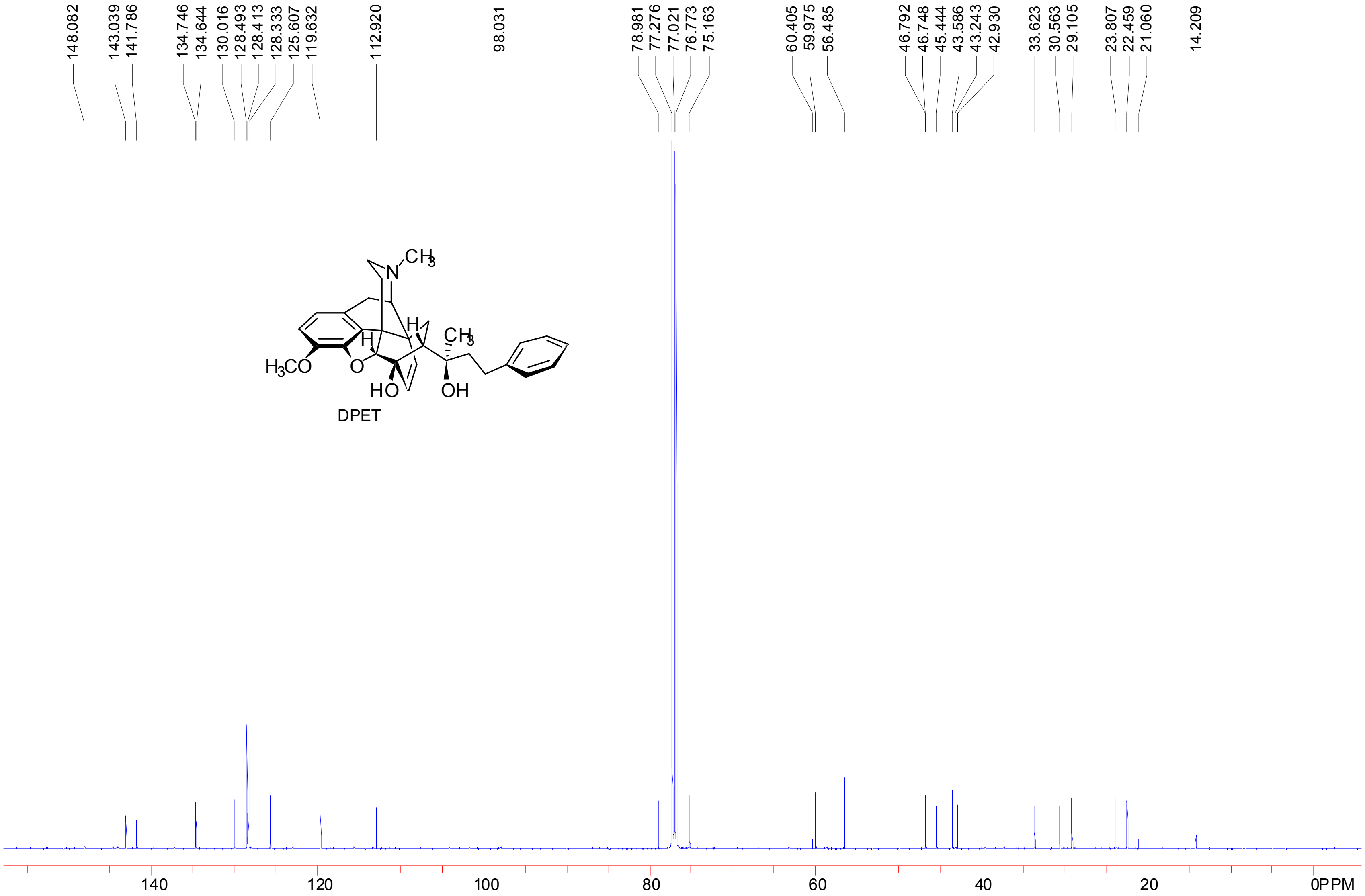

**Figure S23.** ESI-MS of DPET (**9**).

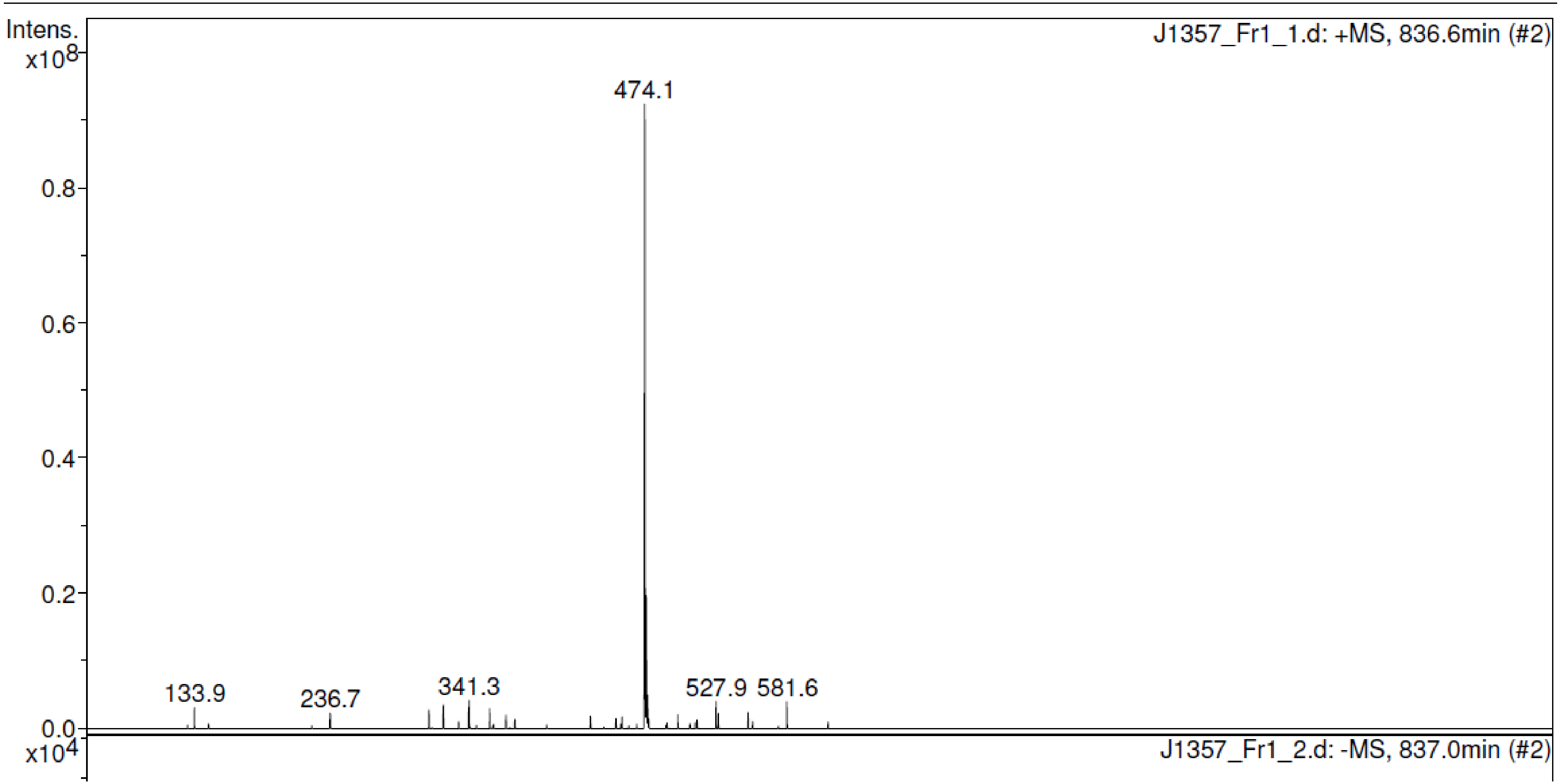

**Figure S24.** $^{1}$H-NMR spectrum of FE-DPET (**10**) in $CDCl_3$.

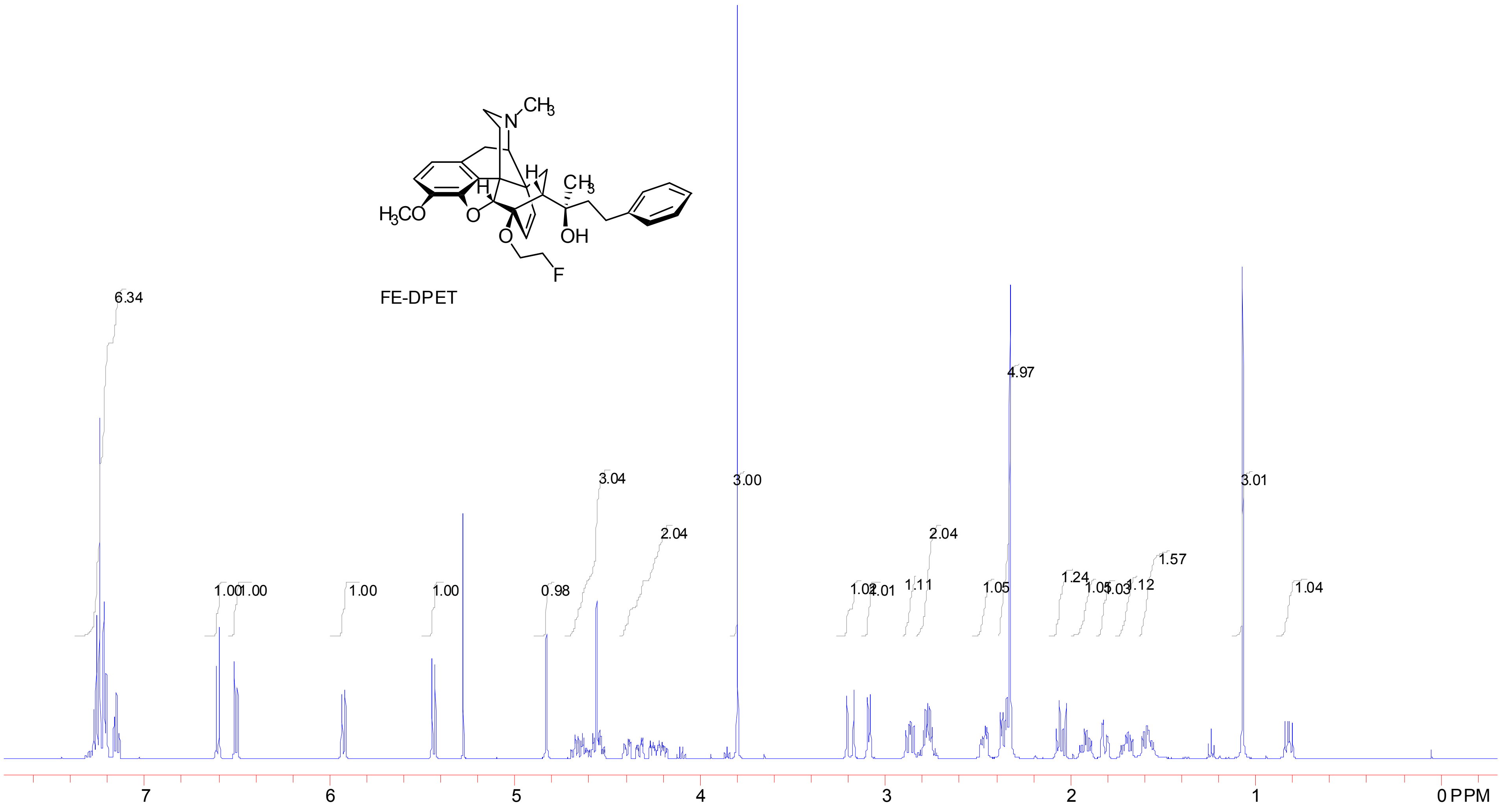

**Figure S25.** $^{13}$C-NMR spectrum of FE-DPET (**10**) in $CDCl_3$.

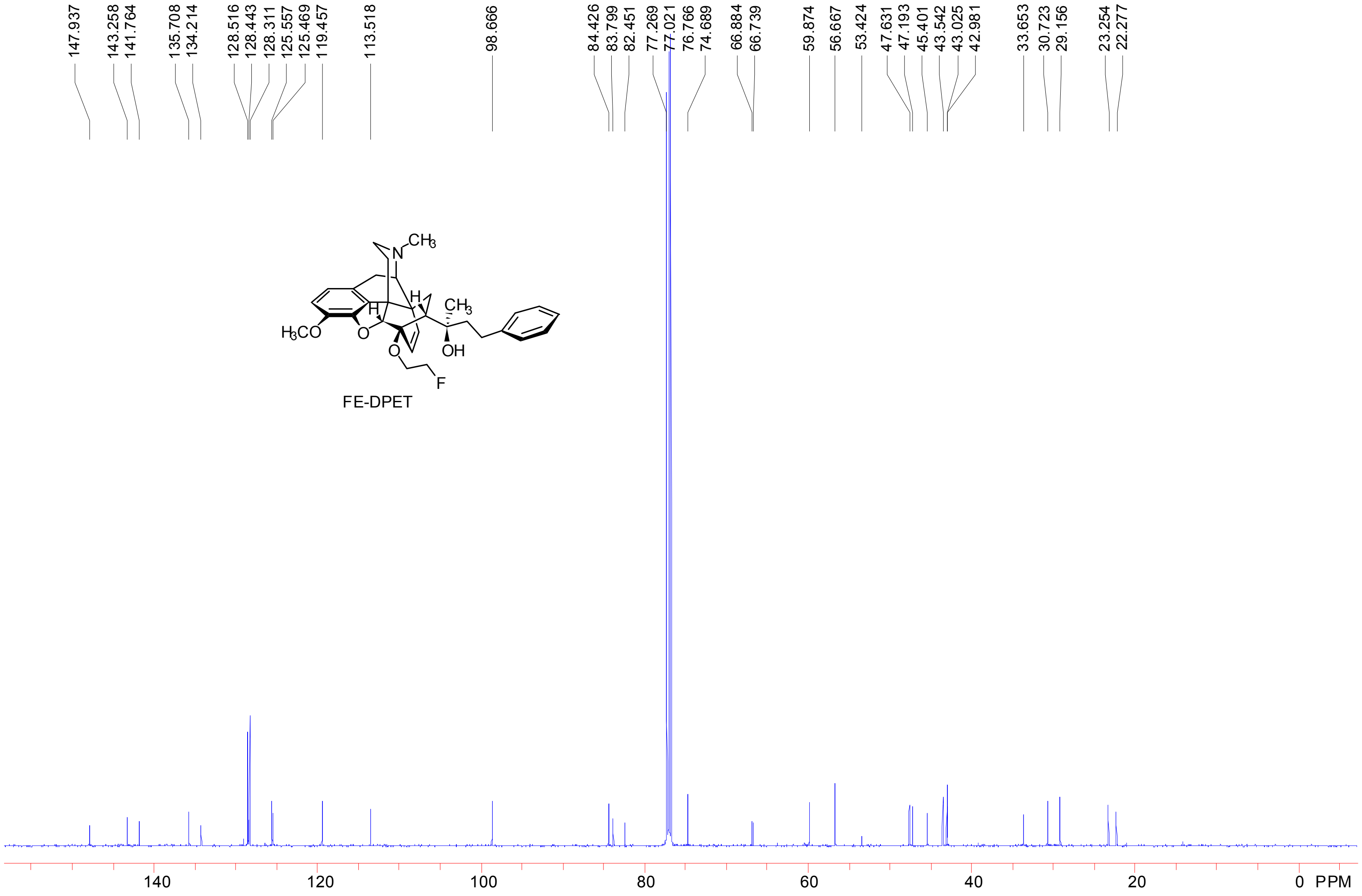

**Figure S26.** $^{19}$F-NMR spectrum of FE-DPET (**10**) in $CDCl_3$.

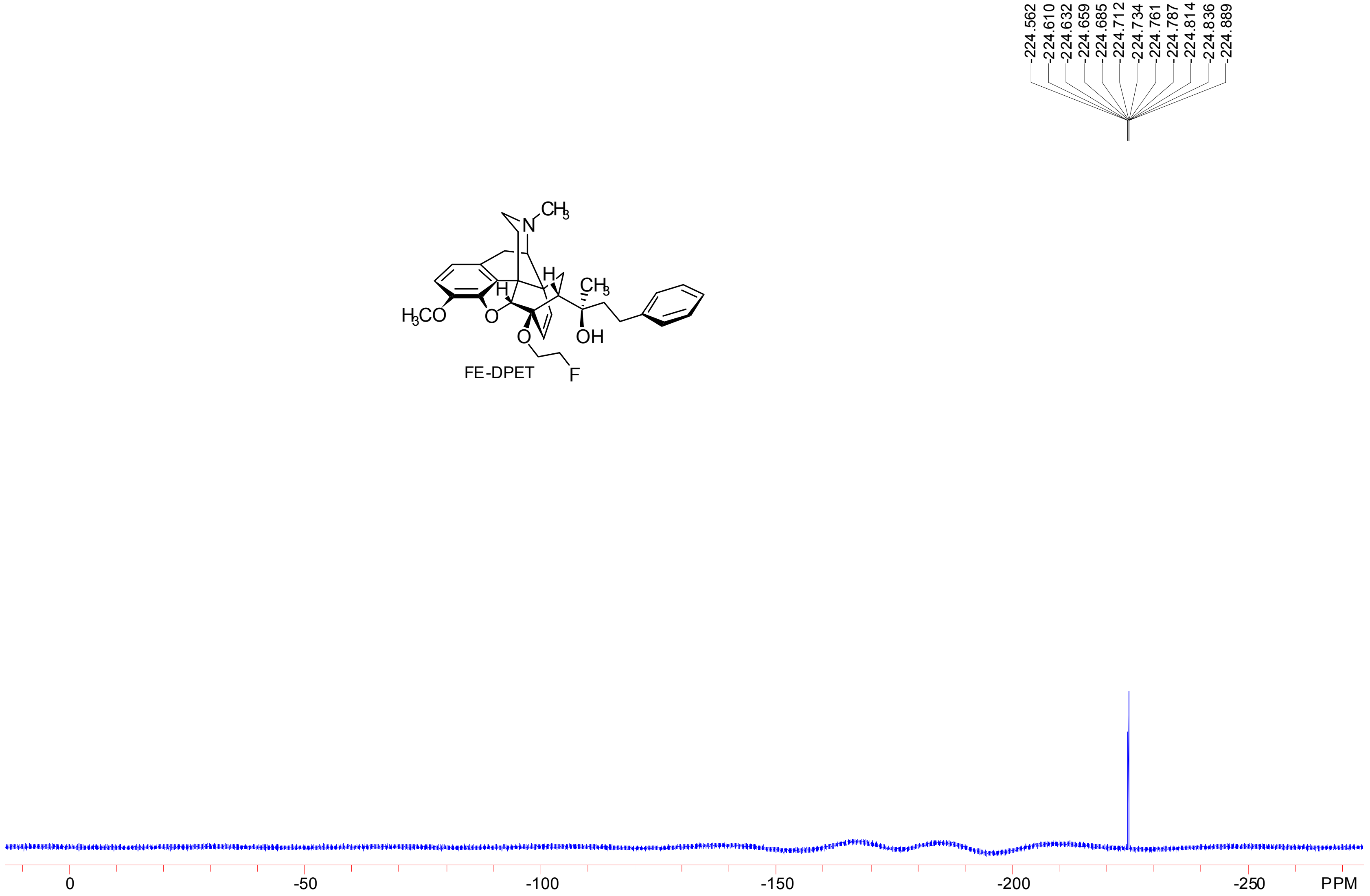

**Figure S27.** ESI-MS of FE-DPET (**10**).

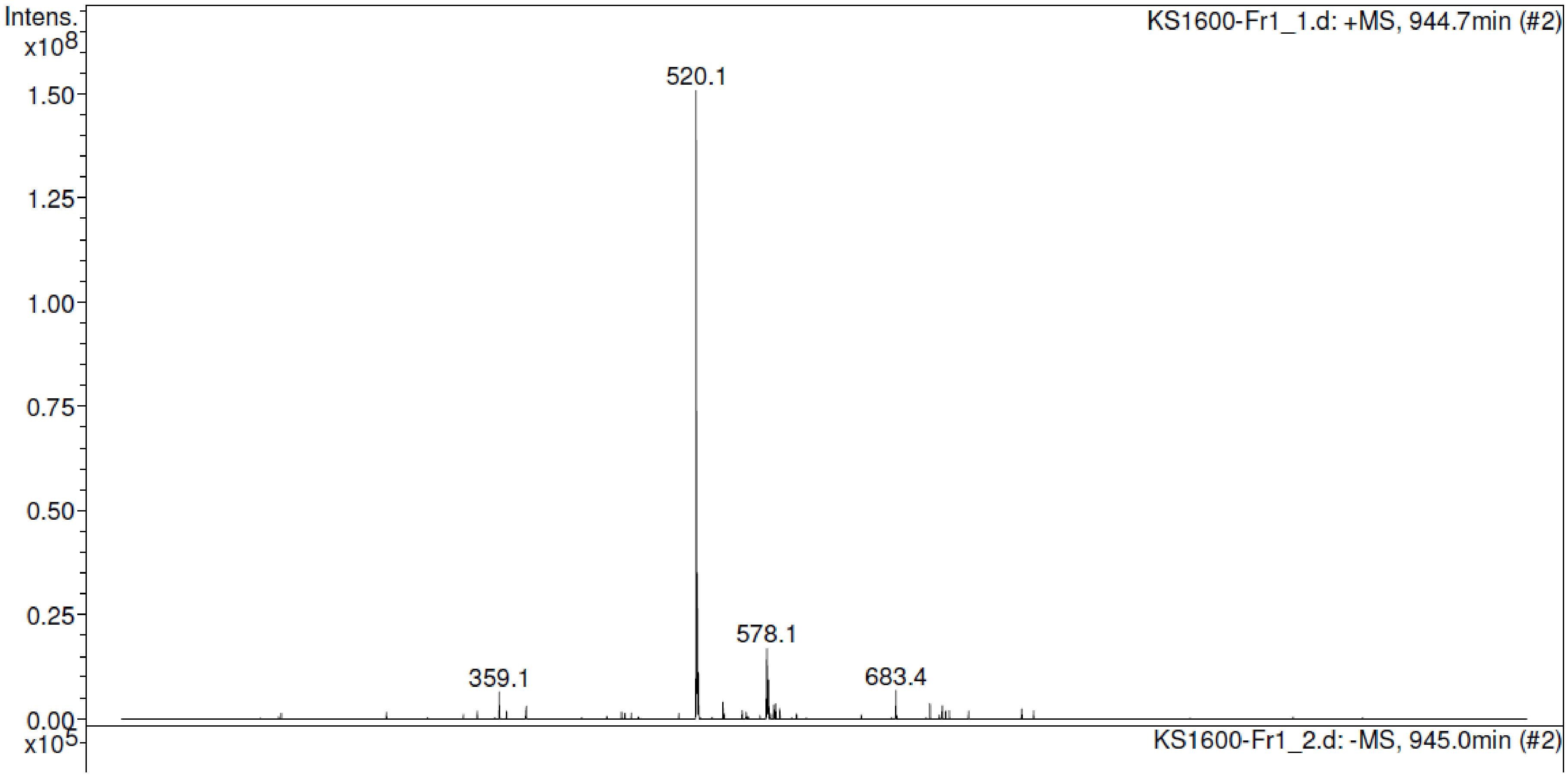

**Figure S28.** $^{1}$H-NMR spectrum of E-DPET (**11**) in $CDCl_3$.

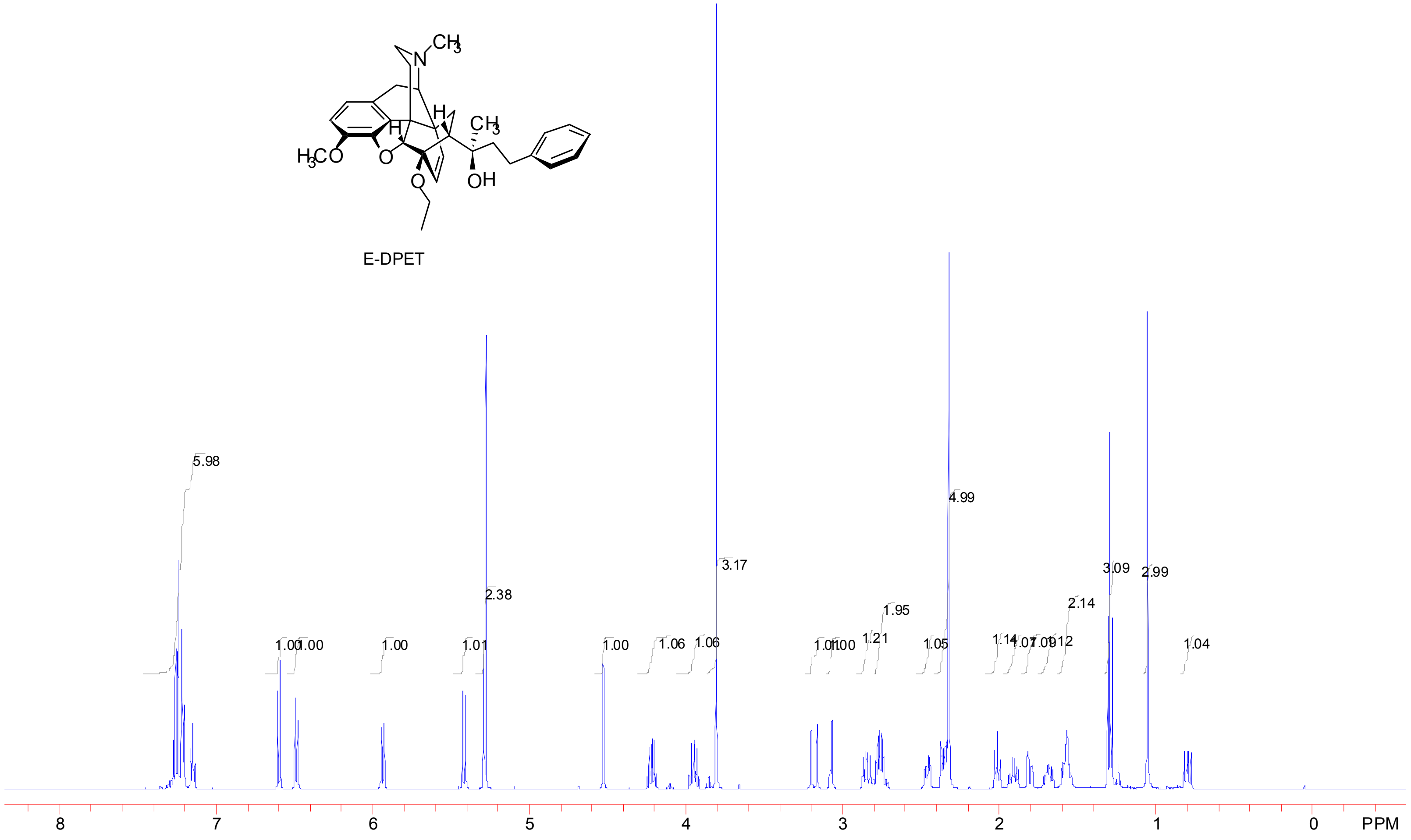

**Figure S29.** $^{13}$C-NMR spectrum of E-DPET (**11**) in $CDCl_3$.

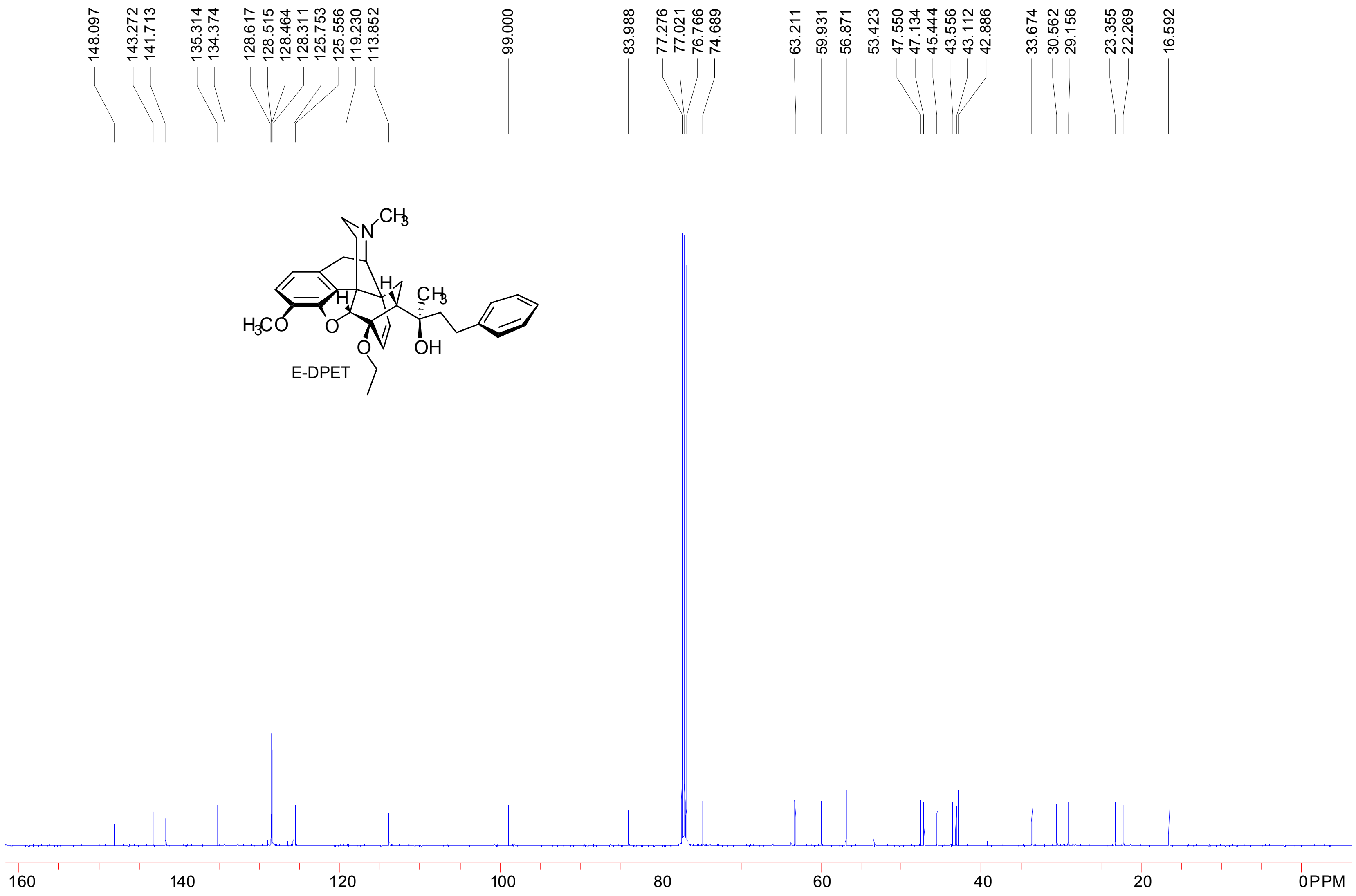

**Figure S30.** ESI-MS of E-DPET (**11**).

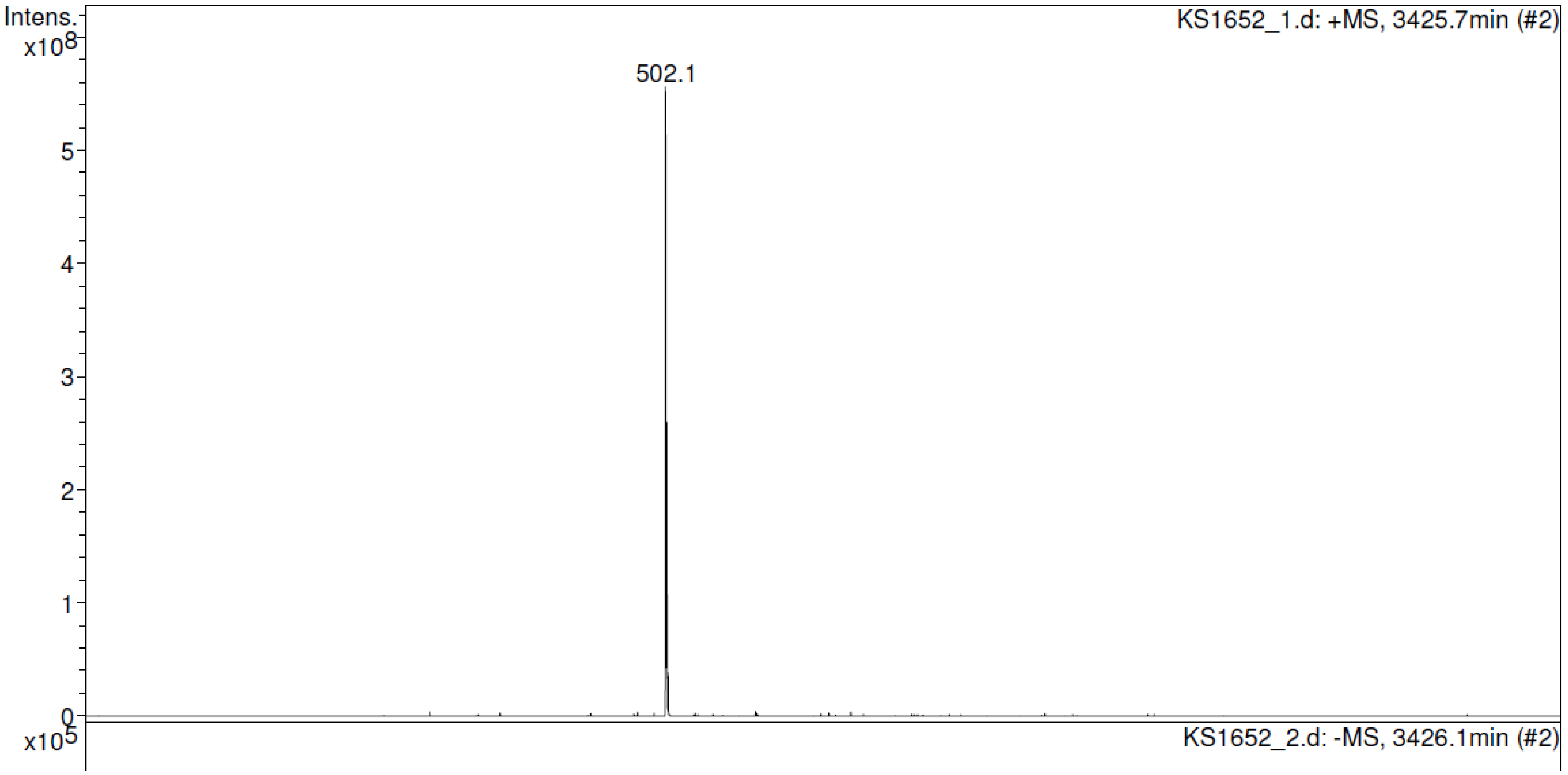

**Figure S31.** $^{1}$H-NMR spectrum of (2-Brom-ethoxy)-*tert*-butyl-diphenyl-silan (**15a**).

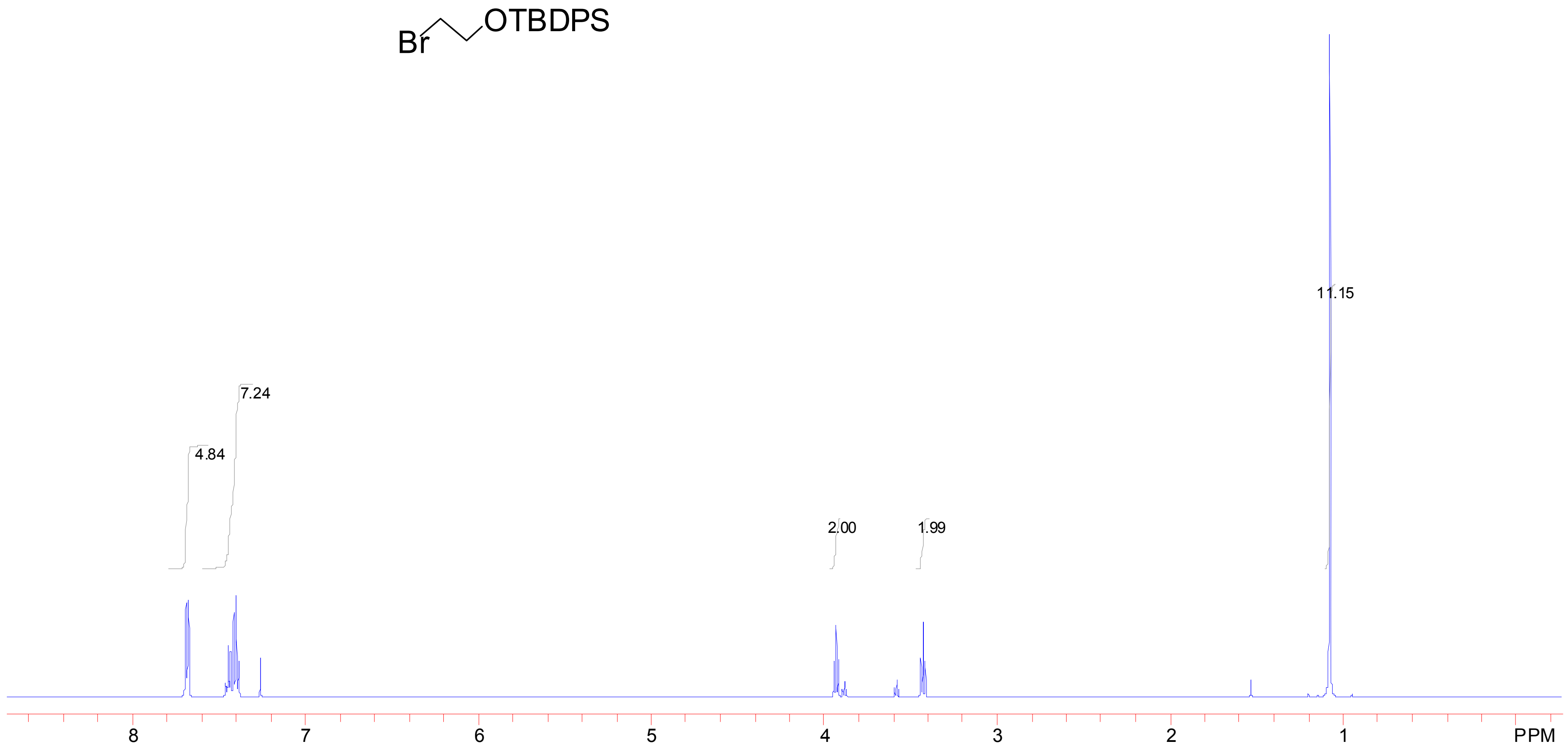

**Figure S32.** $^{13}$C-NMR spectrum of (2-Brom-ethoxy)-*tert*-butyl-diphenyl-silan (**15a**).

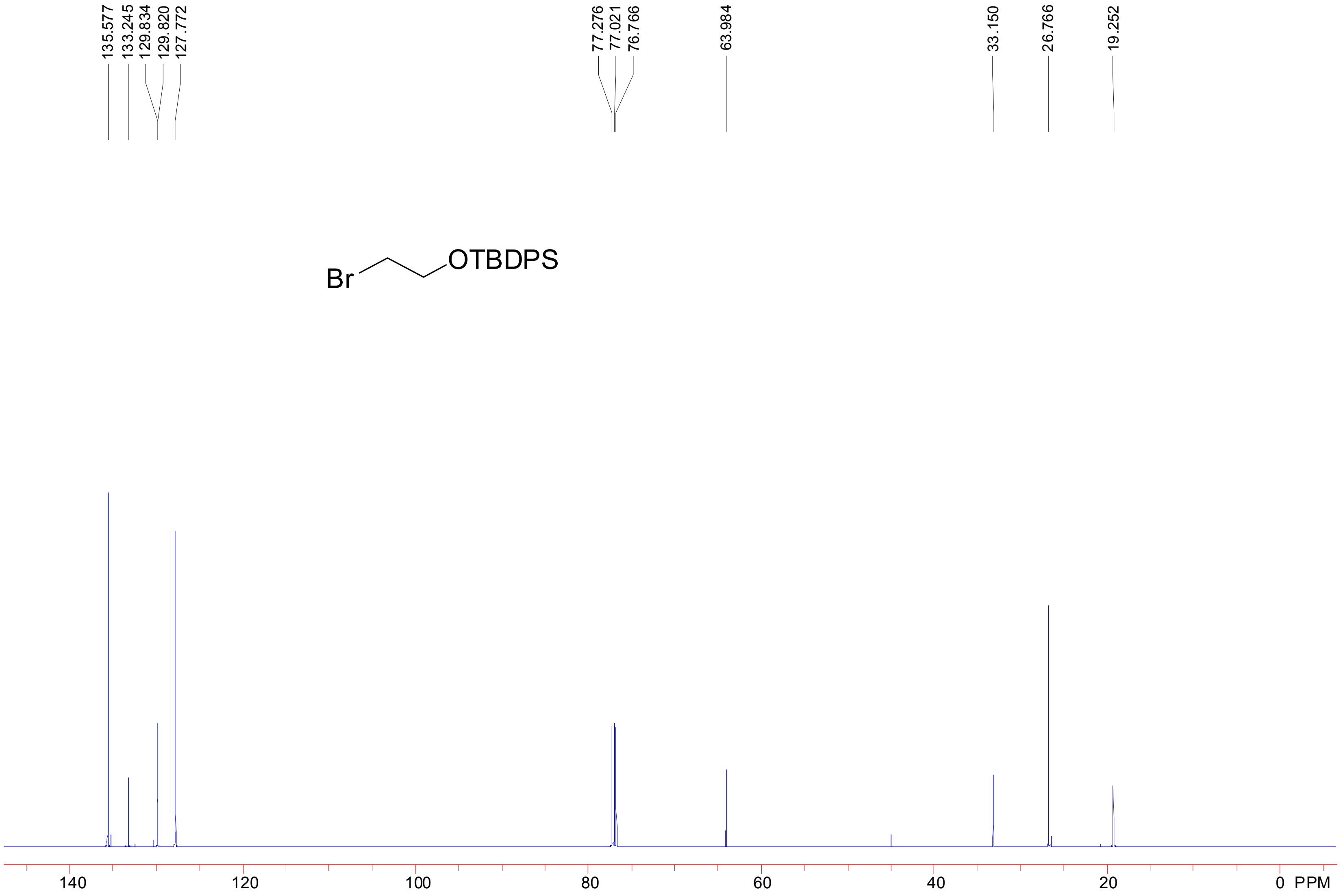

**Figure S33.** $^{1}$H-NMR spectrum of (2-Brom-ethoxy)-*tert*-butyl-dimethyl-silan (**15b**).

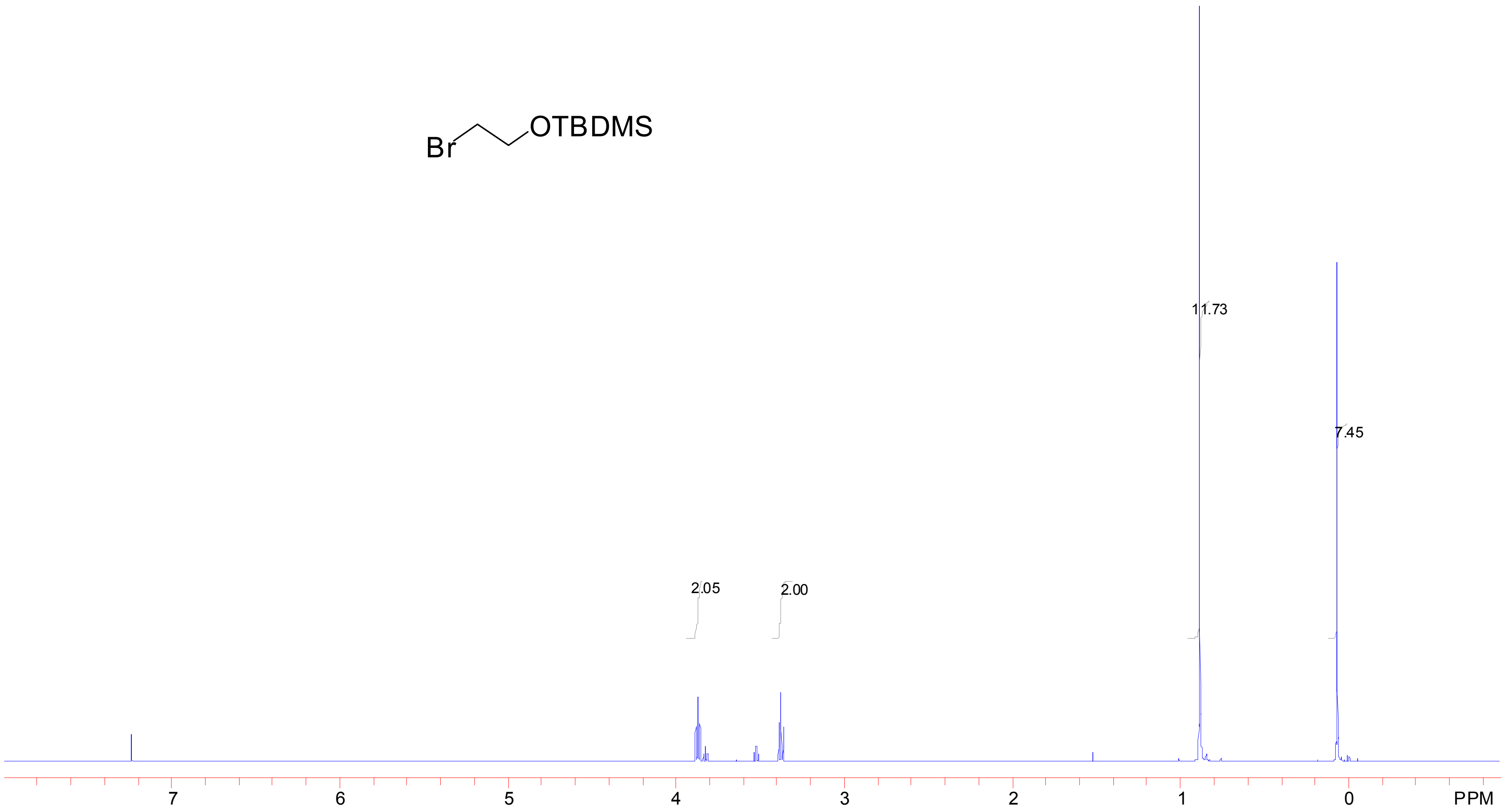

**Figure S34.** $^{13}$C-NMR spectrum of (2-Brom-ethoxy)-*tert*-butyl-dimethyl-silan (**15b**).

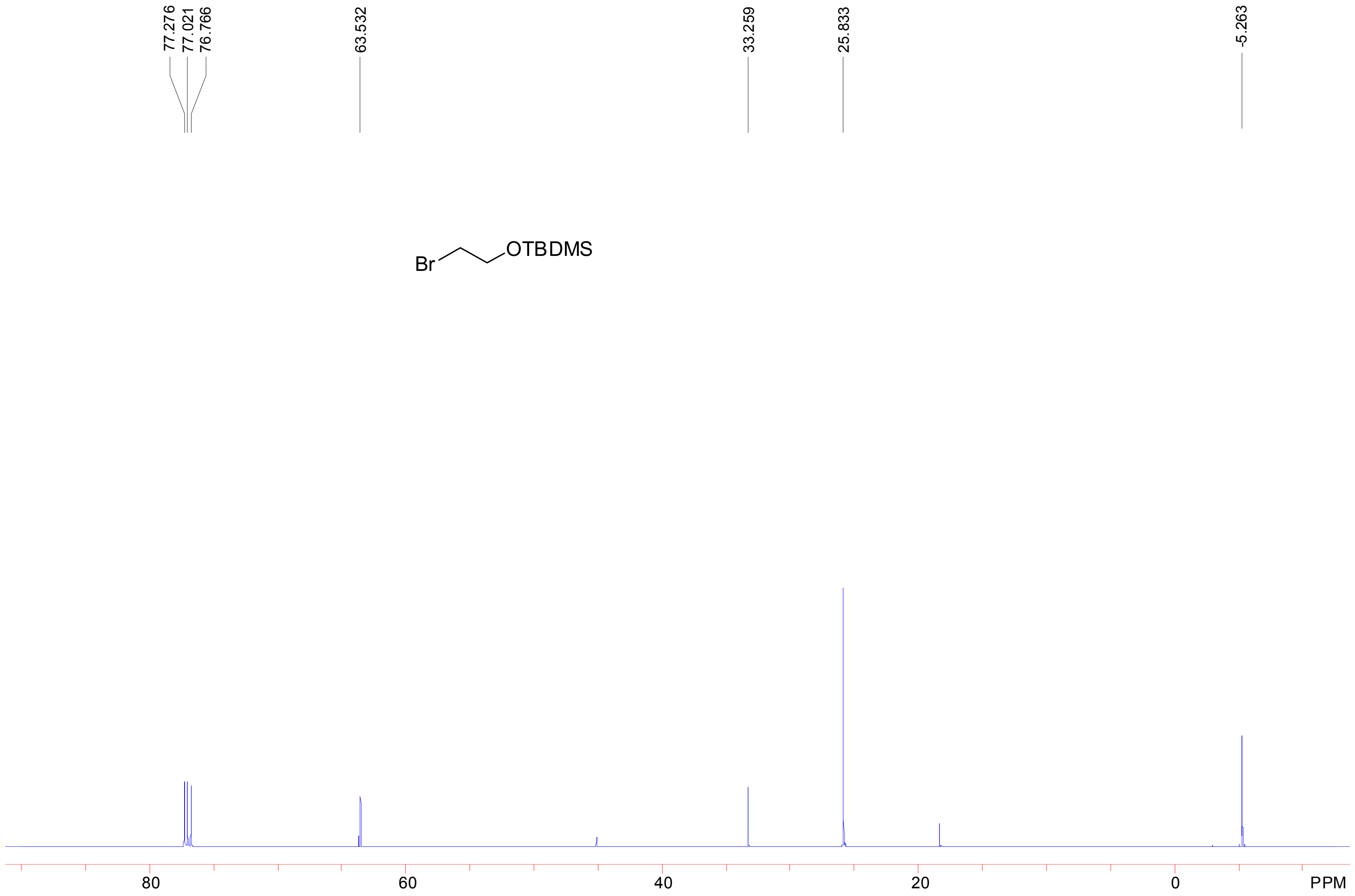

Supplement: Supplementary file 1 [file molecules-17-11554-s001.pdf]
